# Optimized atrazine biodegradation in Egyptian soils via native bacterial isolates and molecular insights using different statistical methods

**DOI:** 10.1038/s41598-026-55623-5

**Published:** 2026-06-16

**Authors:** Abdelaal Shamseldin, Eman S. Ibrahim, Soraya A. Sabery, Hanan Gozalan

**Affiliations:** 1 Environmental Biotechnology Department, Genetic Engineering and Biotechnology Research Institute, at City of Scientific Research and Technology Applications, New Borg El-Arab, Alexandria, Egypt; 2 Botany and Microbiology Department, Faculty of Science, Alexandria University, Alexandria, Egypt

**Keywords:** Atrazine, Egyptian soils, Molecular identification, Atrazine degrading genes, HPLC, Atrazine metabolites, RSM, and CCD, Biological techniques, Biotechnology, Environmental sciences, Microbiology, Molecular biology

## Abstract

Forty four bacterial candidates isolated from Egyptian atrazine polluted soil sites, were examined to select atrazine bio-degraders. Among them thirty four were proved their ability to use it as nitrogen source. Strains HA19 and A7 gave the highest efficient rate to biodegrade atrazine with efficiency of 81% and 81.3%. They were identified and belonging taxonomically to *Klebsiella* sp. and *Ochrobactrum* sp. according to sequencing of 16S rRNA and phylogenetic analysis. Both of them confirmed their possession of atrazine degrading genes like *atzA*, *atzC* and *atzD* while *atzB* was appeared as faint band with strain A7 only. HPLC analysis indicated that the two strains degraded atrazine via the pathway of producing deethylatrazine as an important intermediate. Thus interesting data revealed the possibility of acquisition of these strains for two couples of atrazine degrading mechanisms located on chromosomal and plasmid sites. Applying the advanced statistical method of response surface methodology (RSM) based on central composite design (CCD) model has contributed to increase the biodegradation process with both strains by a rate of (94.2%) and (96.7%) on respectively. For our knowledge this article is one of condensed work and applicable way that will introduce bio safety solution for bioremediating atrazine and reducing its toxicity in agricultural polluted fields, as there is little reports from Egypt published before concerning atrazine bioremediation.

**Supplementary Information:**

The online version contains supplementary material available at 10.1038/s41598-026-55623-5.

## Introduction

Pesticides are synthetic organic materials which having no similar counterpart in the natural world. The majority of them are toxic, having long half-life and resistant to natural biodegradation in the environment. Long times ago, more than thousand products of pesticides are used globally for controlling pest and other diseases. It includes herbicides, insecticides, bactericides and fungicides^1^. Atrazine is one of the famous and widely used herbicides in Egypt to control a large number of plant diseases. Atrazine [2-chloro-4-ethylamino-6-isopropylamino-1, 3, 5-triazine] is used in particularly to control weeds infecting corn and sorghum to avoid the low productivity^1^. Annually 70-90 thousands tone of atrazine is used worldwide, to control against diseases in sugar cane, sorghum, and corn crops^2^. Due to it’s intensively use, relatively long half-life in agricultural soil and moderate mobility in soil, it has been detected in many environmental sites especially in surface water^3^. Penetration of residual particles of atrazine to surface soil and ground water are toxic and carcinogenic for different organisms^4^ such as fishes, algae, insects, aquatic plants, and mammals^5^ and useful microbes like nitrogen fixing rhizobia^6^**.** Due to the toxicity of atrazine which dangerous for human who eat contaminated food, it is necessary to remove it to avoid the harmful effect on human and animals through different applicable methods like bioremediation^7,8^. Bioremediation defined as the use of microorganisms to biodegrade or detoxify pollutants from polluted soil or water. Bioremediation of atrazine has much attention in the last decade as an easy, effective, safety and non-expensive tool to reduce the amounts of pollutants compared to other methods^9–11^.

Many researchers have been revealed that a large number of bacterial genera are capable of biodegrading atrazine as single or consortium strains including *Pseudomonas, Acromobacter* and *Arthrobacter*^12^*, Klebsiella*^13^, *Nocardioides*^14^, *Alcaligenes*^15^*,* as well as fungal strains of *Aspergillus*^16^*, Candida* and *Phaenerochaete*^10^. These atrazine degraders detoxify it by N-de-alkylation or de-halogenations reactions^17^**.**

RSM is an assembly of mathematical and statistical tool that it is useful for the modelling and analysing of problems in which a response of interest is influenced by several variables^18,19^. It can be used to select the best variables to optimize and predict high performance conditions for atrazine degradation^20,21^. One of the tools of RSM is the CCD which is most applicable design that has been used by many researchers worldwide for the optimization process^19,20^. It consists of factorial, axial and central points which help to estimate all possible regression parameters depending on all the obtained results of design points.

Bioremediation using atrazine-degrading bacteria to decontaminate polluted-soil sites is often associated with challenges such as surviving and competing with native microorganisms and to be adopted in soil conditions e.g. pH, temperature, and salinity in order to completely remove this pollutant in these sites. Consequently, it is necessary to select effective microbial strains with high capability of atrazine biodegradation, adapted to survive under different environmental stresses and can utilize the toxic metabolites of intermediates. Several investigators used single or microbial consortium for atrazine biodegradation^12,13,22,23^ and bioremoval of other different pollutants such as heavy metals^24^, and poly aromatic hydrocarbon^25,26^. We aimed in this study to 1; Screen and isolate microorganisms able to use atrazine as nitrogen source from soil-atrazine polluted sites 2; Select the best growth condition for obtaining the highest efficiency of atrazine bioremediation as one variable on time (OVAT) 3; Determining the degradation efficiency of atrazine degrading isolates via its byproducts of metabolites 4; Differentiating and identifying these isolates using molecular techniques 5; Detecting of genes involved in atrazine degradation and comparing their degradation performance between single strain or microbial consortium and 6; Gaining the optimum conditions based on using RSM statistical method and central composite design (CCD) compared to the OVAT.

## Results

### Isolation and characterization of atrazine-degrading bacteria

**Fig. 1:**
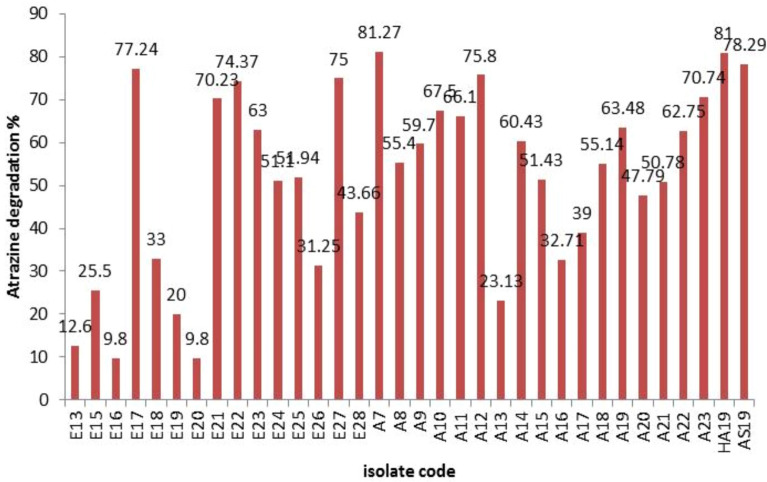
Efficiency values of bacterial isolates for atrazine biodegradation in liquid MSM after 7 days.

Forty-four bacterial isolates were isolated from different polluted Egyptian soils with atrazine using serial dilution method. Strains were screened to test their ability to use atrazine as a main nitrogen source in minimal salt agar media (MSAM) containing a concentration of atrazine up to 1000 mg l^-1^. Thirty four isolates were proved their capability of growing on MSAM with atrazine. The rate of efficiency of atrazine degraders was varied among the examined strains (34) and ranged from 9.8% to 81.8% (Fig. 1). Strains HA19 and A7 gave the highest biodegradation capacity reaching to 81% and 81.3% on respectively (Fig. 1). An additional experiment was done to confirm the ability of these strains to assimilate atrazine as nitrogen source in liquid MSM in the presence of P-aminoacetophenone (PAAP) as substrate to detect atrazine (Supplementary Fig. S1). The yellow colour of polymethine dye indicating for the presence of atrazine in the tubes which it is reduced gradually according to the efficiency of inoculation with atrazine degraders compared to the high resolution of yellow colour of non–inoculated control.

### Morphological and biochemical characteristics of atrazine degraders

The morphological observations of colony from strain HA19 was 2-3mm, translucent-opaque, mucoid, circular, dome-shaped with soft and milky texture and slight shiny, while strain A7 was white to beige, mucoid with entire edges and 2–3 mm in diameter on LB agar medium. Strain HA19 grew rapidly on LB solid plates (Supplementary Table S1). Both of the two strains HA19 and A7 were short rods, Gram negative and non-motile spore forming. Both of them were able to assimilate lysine, ornithine, glucose, mannitol, citrate and TDA. Strain HA19 was able to use lactose but strain A7 was not. Strain A7 could assimilate xylose while another strain failed. The two strains were negatively produce indole but positively could reduce nitrate (Supplementary Table S1).

### Identification of atrazine degraders using molecular tools

**Fig. 2:**
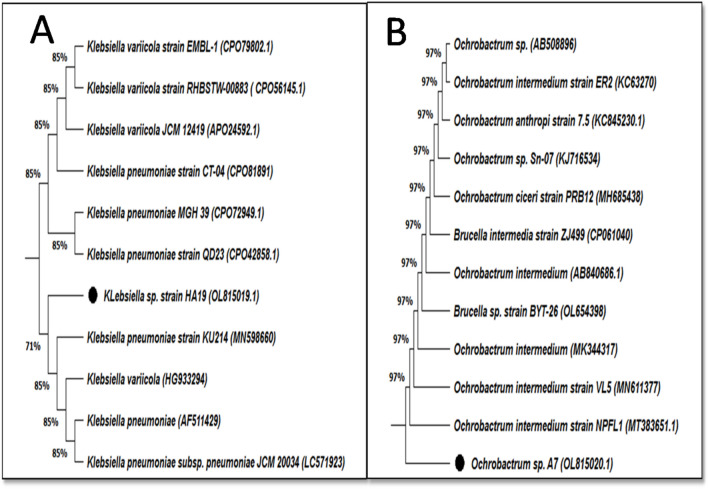
Phylogenetic tree based on 16S rRNA gene sequence analysis using neighbour-joining method. The Atrazine degrading isolates (**A**) *Klebsiella sp.* HA19 and (**B**) *Ochrobactrum* sp. A7 are remarked by black dots.

The DNA from strains HA19 and A7 was used as a template with specific primers and PCR conditions to amplify the 16S rRNA. An amplified fragment of 1500 bp was generated from the two examined strains (Data not shown). The generated fragment from each strain subjected to sequencing and their sequences assembly of 16S rRNA of both strains compared with the same gene from several available strains on the NCBI server using BLAST tool (https://blast.ncbi.nlm.nih.gov/Blast.cgi). Data revealed that strain HA19 was closely related to *Klebsiella* sp. with similarity of 99.5%, while strain A7 was closely related to *Ochrobactrum* sp*.* with 99.7% similarity (Supplementary Table S2). Phylogenetic trees based on 16S rRNA sequences were reconstructed **(**Fig. 2**)**, to confirm the genetic affiliation of these two strains to the two genera. Results of phylogenetic trees indicated that strain HA19 shared the ancestral clade with strain *Klebsiella pneumoniae* MGH 39 with 93% identity. On the other side strain A7 gave unique clade more closely related to genetic lineage of *Ochrobactrum intermedium* strain NPFL1*.* Principally, strain HA19 identified as *Klebsiella* sp*.* and strain A7 identified as *Ochrobactrum* sp*.*

### Growth curves of the two strains during atrazine degradation

The growth curves of both of *Klebsiella* sp*.* and *Ochrobactrum* sp*.* showed that the two strains HA19 and A7 entered the logarithmic phase after 24h with low rate of atrazine degradation scored 6.09 and 4.07 mg l^–1^ on respectively (Supplementary Fig. 2). Strain HA19 of *Klebsiella* sp*.* gave the highest growth after 4 days with an OD of 1.205 at 600 nm which associated with degradation rate up to 43.42%. Strain A7 of *Ochrobactrum* sp. reached the highest level of growth after 3 days with an OD 600 of 1.628 reflecting 30.9% atrazine degradation. Then the two strains entered in the decline phase obviously. After 6 days atrazine degradation increased during the decline phase and reached up to 64% and 69.8% with both of them (Supplementary Fig. S2).

### Scanning electron microscopy (SEM) of treated cells with atrazine

**Fig. 3:**
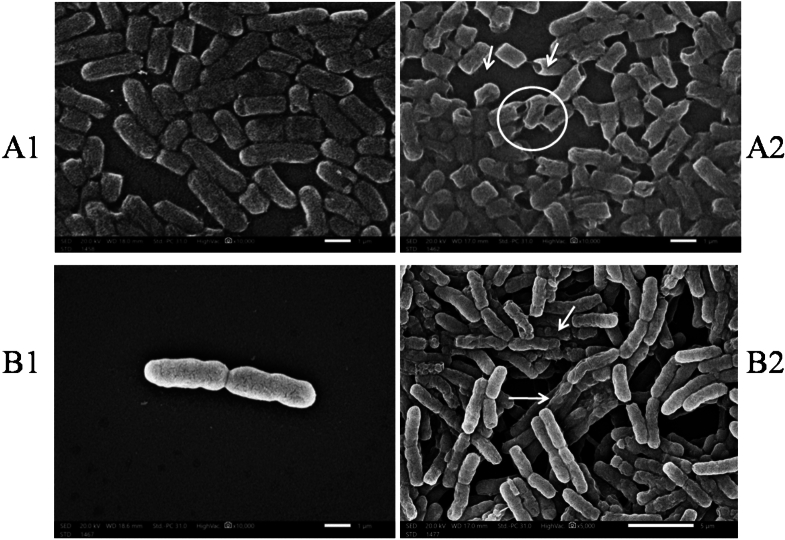
Morphological changes by scanning electron microscopy. (**A1**) *Klebsiella* sp. HA19 normal cells, (**A2**) Cells of *Klebsiella* sp. treated with 100ppm of atrazine and (**B1**) *Ochrobactrum* sp. A7 normal cells, (**B2**) Cells of *Ochrobact*rum sp. treated with 100 ppm of atrazine. Arrows and circles indicate the distortion.

The attitude of strain HA19 and A7 with atrazine degradation_,_ and the subsequent changes in cell morphology examined using SEM. The untreated strain of HA19 appeared normal with healthy shape as smooth, short-rods with uninjured surface morph structure (Fig. 3A1, A2). In contrast, cells of this strain appeared cracked/damaged, distorted, misshaped and somehow decreased in length due to presence of atrazine. Regarding to strain A7*,* the cells appeared as intact single or diploid long-rods with no evidence of cell wall rupture and collapse before treating with atrazine (Fig. 3B1, B2). After expose to atrazine cells, strain A7 had less destructive effect, some cells kept its original length and shape, but some other cells became coarser with groove-like rifts on the surface and the cell morphology was changed noticeably.

### Detection of atrazine degrading genes

**Fig. 4:**
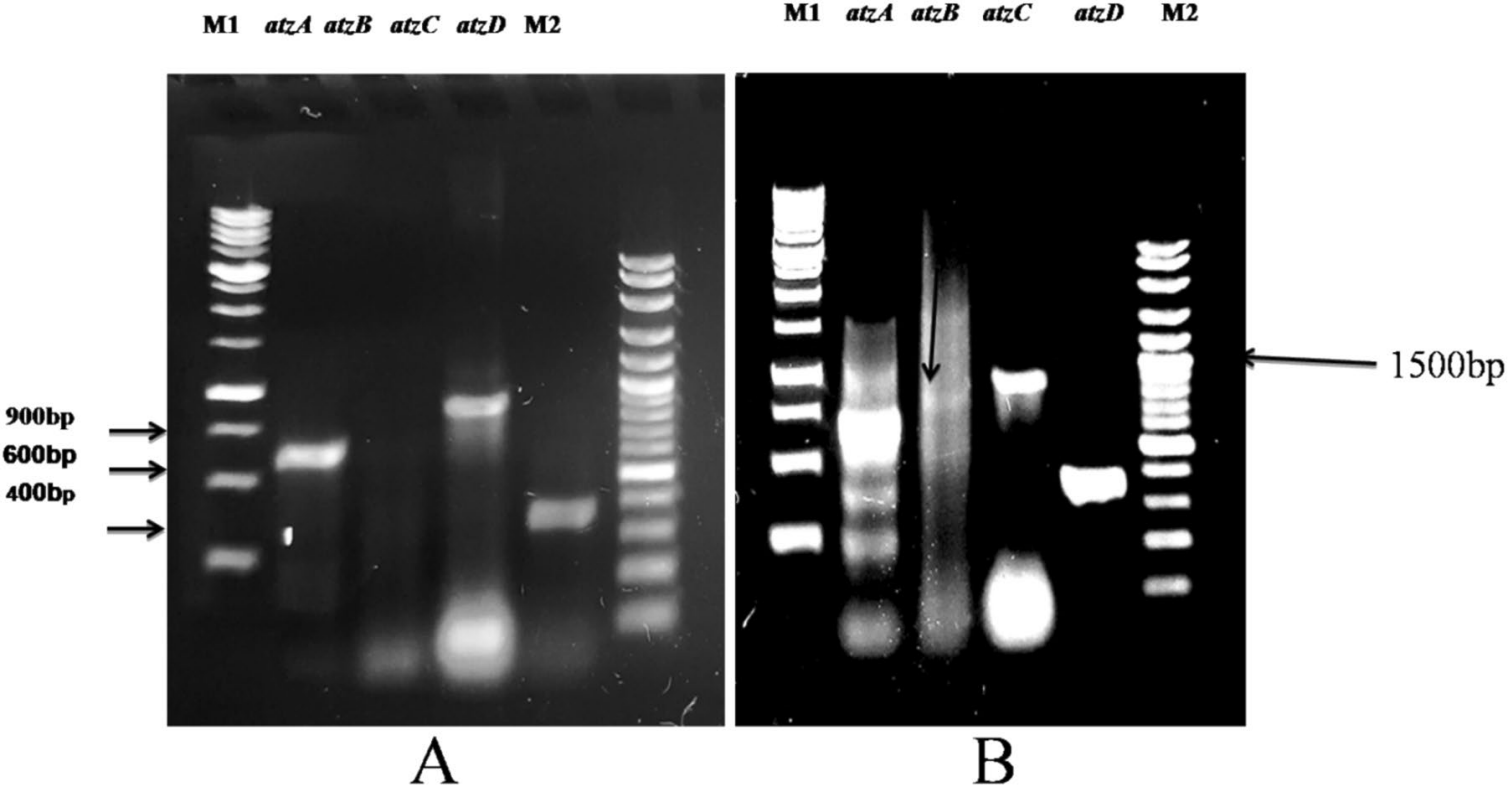
Agarose gel electrophoresis of atrazine degrading genes for strain *Klebsiella* sp. HA19 (**A**) and Ochrobactrum sp A7 (**B**), *atzA, atzB, atzC, atzD*, M1,1Kb DNA ladder RTU, M2, VC 100bp plus DNA ladder

Both strains were screened for the presence of DNA homologous to the *Pseudomonas* strain ADP *atzABCD* genes, which encode atrazine degradation enzymes. Results obtained from PCR experiments to amplify genes (*atzA, atzB, atzC* and *atzD*) from strains HA19 (Fig. 4A) and A7 were shown in (Fig. 4B). Three degrading genes *atzA, atzC*, and *atzD* were successfully amplified in the two examined strains but *atzB* gene fragment was detected as a weak band only with strain A7. The PCR yield of *atzA* of strain HA19 and A7 detected at 600 bp and 500 bp, while other fragments of *atzC* and *atzD* were appeared at about 900 bp and 400 bp in both tested strains, on respectively.

### HPLC analysis to detect metabolites of atrazine after degradation

**Fig. 5:**
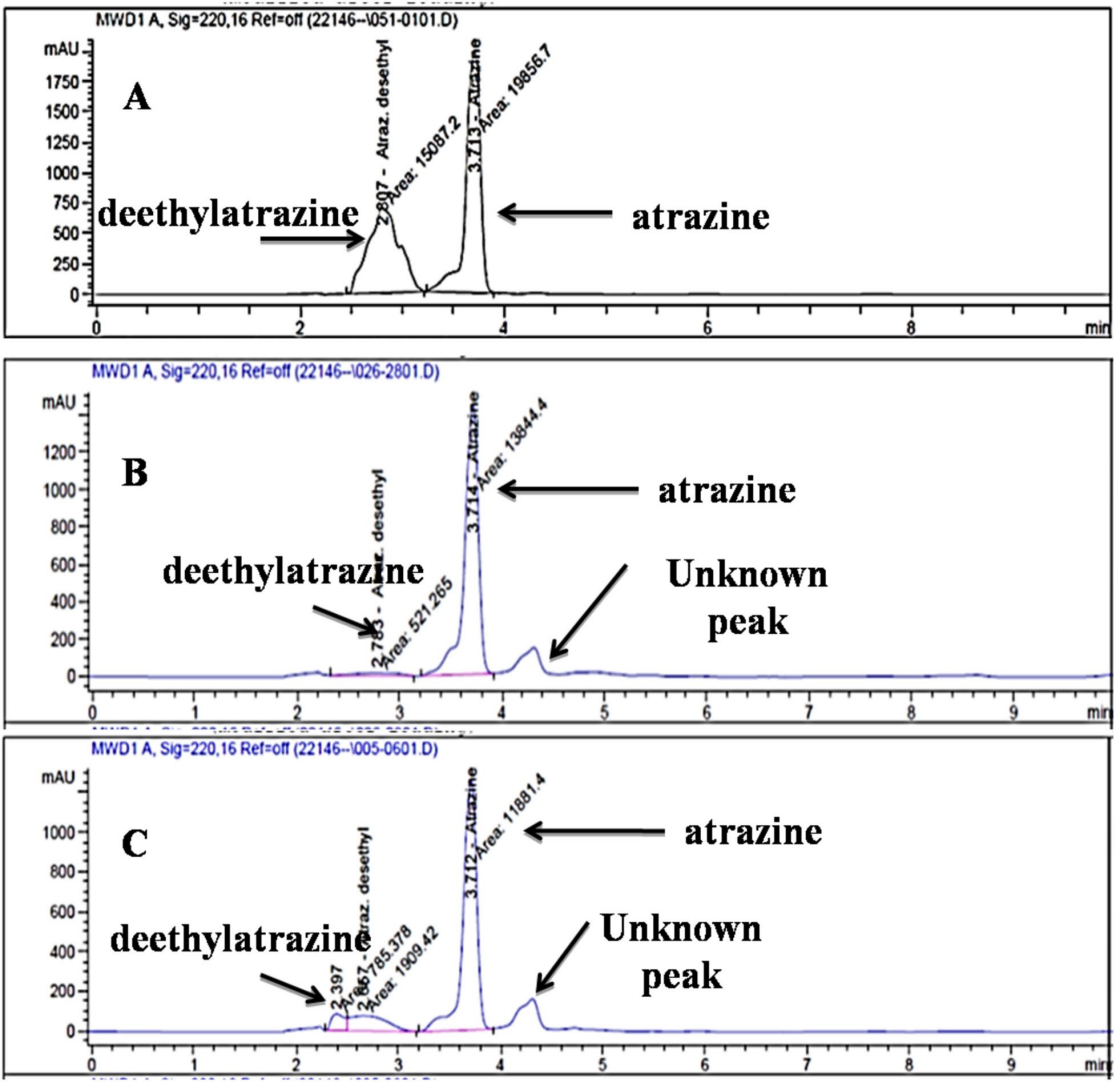
HPLC chromatograms of atrazine degradation; (**A**) atrazine and deethylatrazine standard. (**B**) atrazine degradation and deethylatrazine production by *Klebsiella* sp. HA19 and (**C**) *Ochrobactrum* sp. A7, on respectively.

**Fig. 6:**
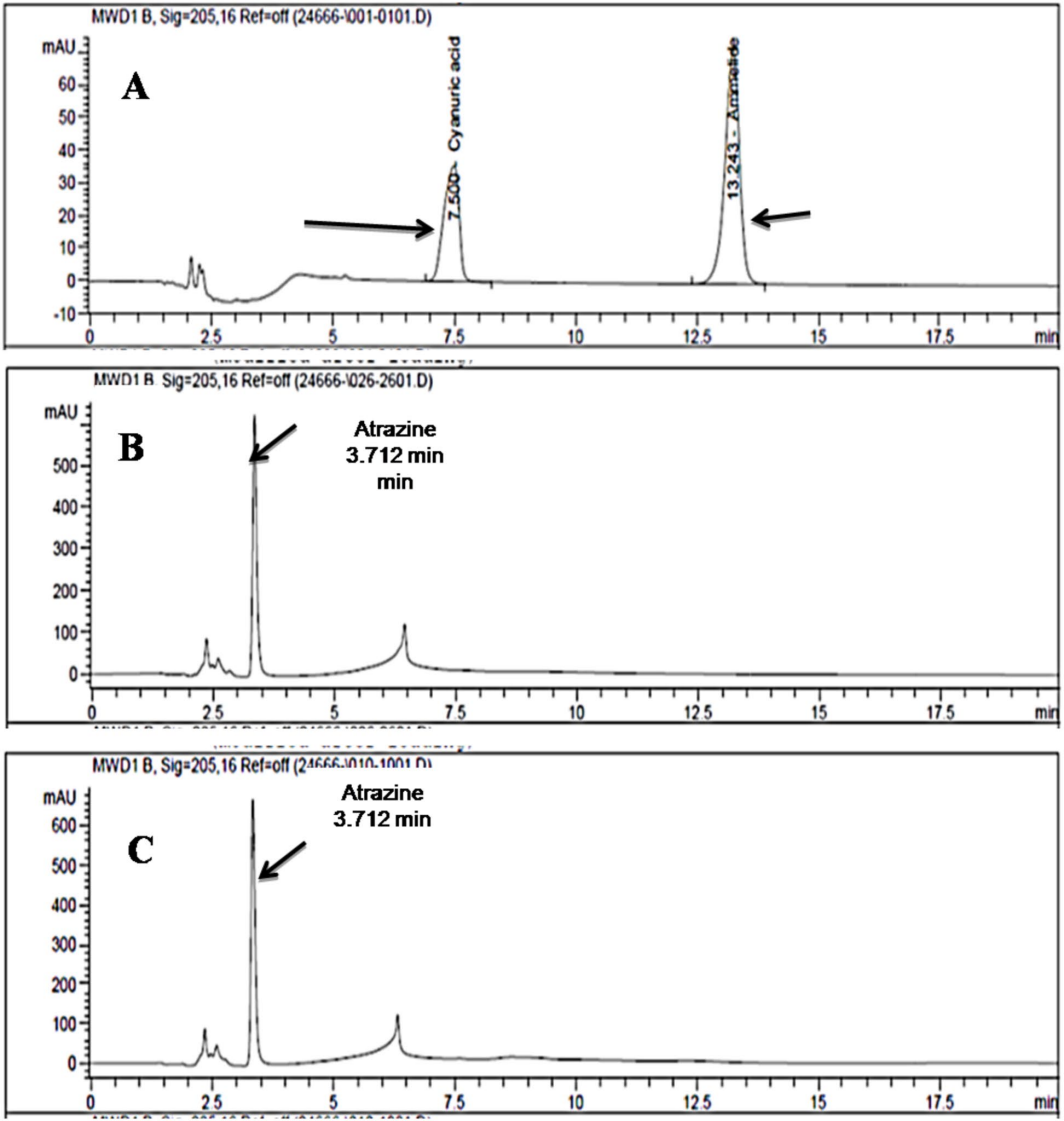
HPLC chromatograms of cyanuric acid and ammelide standard (**A**). No detection of cyanuric acid and ammelide by both strains of *Klebsiella* sp. HA19 (**B**) and: *Ochrobactrum* sp. A7, (**C**) on respectively.

The results of HPLC (Fig. 5 A, B, C) indicated that the interested two strains were degrading atrazine efficiently and the intermediate deethylatrazine (DEA) was detected at retention time of 2.807 min with both strains. Its concentration increased first until reached the highest level after the fifth day (1.31 mgl^-1^and 2.91 mgl^-1^) by strains HA19 and A7 on respectively. An unknown additional peak at retention time of 3.3 min was noticed by the two strains. In our results we could not detect both of the two intermediates cuynaric acid and ammelide (Fig 6A, B, C).

### Estimation of deetylatrazine (DEA) in liquid culture for both strains

**Fig. 7:**
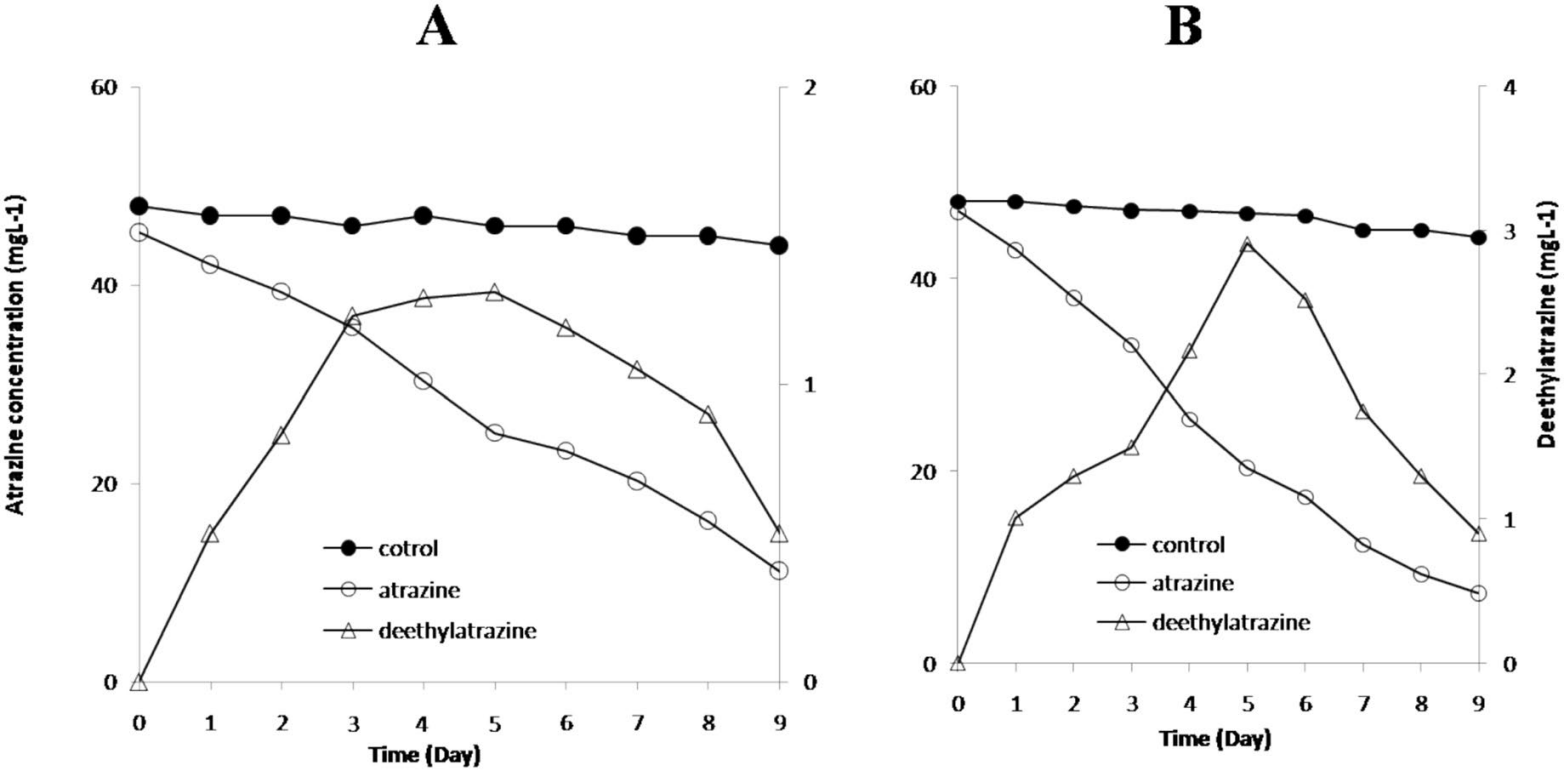
Concentration of atrazin and deethylatrazine by (**A**) *Klebsiella* sp. HA19 and (**B**) *Ochrobactrum* sp. A7after exposure to 50ppm atrazine

Physiological results in (Fig 7A, B) indicated that the two strains were degrading atrazine efficiently. The degradation efficiency of 50 mgl^-1^ atrazine by *Klebsiella* sp. HA19 reached about 75.2% (11.7mgl^-1^ of atrazine was remained) after 10 days, while that of *Ochrobactrum* sp. A7 reached 84.4% (7.31 mgl^-1^ of atrazine was remained). *Ochrobactrum* sp. strain A7 was higher efficient for atrazine degradation than *Klebsiella* sp. HA19*.* DEA concentration increased first until reached the highest concentration after the fifth day (1.31mgl^-1^and 2.91mgl^-1^) by strains *Klebsiella* sp. HA19 and *Ochrobactrum* sp. A7, (Fig 7A, B) on respectively.

### Bioaugmentation of atrazine degradation in environmental samples (soil and agricultural effluents)

**Fig. 8:**
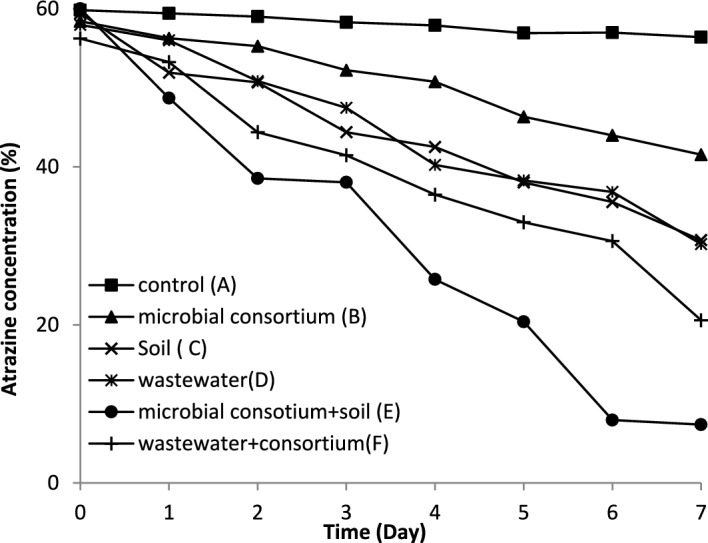
Residual atrazine in Minimal Salt Medium (MSM) with treatments; (**A**) inoculum free MSM, (**B**) MSM+ microbial consortium, (**C**) MSM+ unsterilized natural soil (**D**) unsterilized agricultural waste water (**E**), MSM+ microbial consortium + unsterilized natural soil, and (**F**), unsterilized agricultural waste water + microbial consortium

Bio-augmentation with a simple or microbial consortium of strain HA19 *Klebsiella* sp and A7 *Ochrobactrum* sp. was used for bioremediating atrazine contaminated soil and agricultural waste water. The degradation efficiency of atrazine (100 mg^-1^) by microbial consortium reached 41.54% and 29.9% in soil and in agricultural waste water, respectively (Fig. 8) after seven days while the degradation effectiveness of individual indigenous microbial agents of soil and agricultural waste water reached 48.88% and 50.4% respectively (Fig. 8). When the microbial consortium was mixed with soil and waste water for the degradation of atrazine, the degradation activity could be significantly enhanced and reached 87.68% for soil and 66.25% for waste water. Therefore, the ability of enrichment culture to degrade atrazine in unsterilized soil and agricultural waste water indicated that atrazine degrading consortium was able to survive and multiply even in the presence of native soil micro-flora.

### Atrazine bioremediation using RSM analysis based on CCD model

Experiments were performed to study the combined effect of four different parameters (independent variables) on atrazine degradation (response). A five-level (–*a*, –1, 0, +1, + *a*) CCD (Supplementary Table S3) with 4 independent variables (inoculum size, trace elements, glucose and atrazine concentrations) were applied in this study in 31 sets of experimental runs consisting of 16 factorial (cubic points), 8 axial (star points ) that are, points having an axial distance to the centre of (*a =±* 2), and 7 replicates of centre points, as the risk of missing non-linear relationships in the middle of the intervals has to be minimized and the repetition allows determining confidence intervals.

Minitab 15.0 (Minitab Inc., Pennsylvania, USA) software was used to determine the second order polynomial coefficients for each term of the equation through multiple-regression analysis. All the experimental and predicted values of atrazine degradation percentages by *Klebsiella* sp. HA19 and *Ochrobactrum* sp. A7 were indicated in Supplementary Table S4 and Table S5, respectively. The maximum degradation percentage (94.2%) by *Klebsiella* sp. HA19 was observed in factorial experiment number 25 with glucose concentration 1gl^-1^ (X_1_ = -1), atrazine concentration 150 ppm (X_2_ = 1), inoculum size 10% (X_3_ = 1) and trace element 3ml (X_4_ = -1), while the minimum degradation percentage (67.9%) was observed in factorial experiment number 21 with glucose concentration 1gl-1 (X_1_ = -1), atrazine concentration 50 ppm (X_2_ = -1), inoculum size 10% (X_3_ = 1) and trace element 10ml (X_4_ = 1). Regarding to *Ochrobactrum* sp. A7, the maximum degradation percentage (96.7%) was observed in axial experiment number 20 with glucose concentration 2.5gl^-1^ (X_1_ = 0), atrazine concentration 200 ppm (X_2_ = 2), inoculum size 5% (X_3_ = 0) and trace element 5 ml (X_4_ = 0), while the minimum degradation percentage (69%) was observed in axial experiment number 18 with glucose concentration 2.5 gl^-1^ (X_1_ = 0), atrazine concentration 25 ppm (X_2_ = -2), inoculum size 5% (X_3_ = 0) and trace element 5ml (X_4_ = 0).

### Numerical analysis of atrazine degradation. Experimental design and quadratic regression models for atrazine degradation

The predicted regression equations provided valuable information about the turning process which indicated on the effectiveness of factors individually and interactively. According to the experimental results, the mathematical model correlates the atrazine degradation percentage by *Klebsiella* sp. HA19 with independent process variables is:

1 $$\begin{aligned} Y \left(atrazine\ deg. \%\right)\hspace{0.17em}&=\hspace{0.17em}80.12\hspace{0.17em}+\hspace{0.17em}1.0792 X_{1}\hspace{0.17em}+\hspace{0.17em}4.1542 X_{2}\hspace{0.17em}+0.3375 X_{3}-2.92 X_{4}\hspace{0.17em}\\&+\hspace{0.17em}0.8939 X_{{1}^{2}}\hspace{0.17em}+\hspace{0.17em}1.0439 X_{{2}^{2}}\hspace{0.17em}+\hspace{0.17em}1.6689 X_{{3}^{2}}\hspace{0.17em}+0.534 X_{{4}^{2}}\\&-0.6938 X_{1}X_{2}\hspace{0.17em}+\hspace{0.17em}1.8812 X_{1}X_{3}\hspace{0.17em}+\hspace{0.17em}3.0312X_{1}X_{4}\hspace{0.17em}+0.6187X_{2}X_{3}\\&-0.6812X_{2}X_{3}-1.8313X_{3}X_{4} \end{aligned}$$

But for strain *Ochrobactrum* sp. A7 is:

2 $$\begin{aligned}Y \left(atrazine\ deg. \%\right)\hspace{0.17em}&=\hspace{0.17em}82.85\hspace{0.17em}+\hspace{0.17em}0.5167 X_{1}\hspace{0.17em}+\hspace{0.17em}5.5083 X_{2}\hspace{0.17em}+0.6 X_{3}\\&- 0.2083X_{4}\hspace{0.17em}+\hspace{0.17em}0.3649 X_{{1}^{2}}\hspace{0.17em}+\hspace{0.17em}0.3024 X_{{2}^{2}}\hspace{0.17em}\\&+\hspace{0.17em}0.8399 X_{{3}^{2}}\hspace{0.17em}+0.9774 X_{{4}^{2}}- 1.025 X_{1}X_{2}\hspace{0.17em}\\&+\hspace{0.17em}0.45 X_{1}X_{3}\hspace{0.17em}+\hspace{0.17em}1.35 X_{1}X_{4}\hspace{0.17em}+0.85X_{2}X_{3}\\&-0.9X_{2}X_{3}- 0.4 X_{3}X_{4}\end{aligned}$$

Where, Y is the atrazine degradation %, X_1_, X_2_, X_3_, and X_4_ are the coded values of the test variables, glucose concentration (gl^-1^), atrazine concentration (ppm) inoculum size (v/v) and trace elements (v/v), respectively.

### Effects of influencing factors on atrazine degradation

The estimated regression coefficients and *p*-values of linear, quadratic and interaction levels of the independent variables affecting on atrazine degradation by *Klebsiella* sp. HA19 and *Ochrobactrum* sp. A7 were shown in Supplementary Table S6 and Table S7, respectively. In the case of *Klebsiella* sp. HA19, it was observed that glucose was positive but non-significant factor at linear (A) and quadratic (A*A) levels while atrazine was positive and significant at linear (B) level meaning that the change of its concentration would influence on atrazine degradation.

For the interaction term, the interactions between glucose and inoculum size (A*C) ;glucose and trace elements (A*D) were found to be positive significant as given by *p*-values <0.05, while the interaction between atrazine and inoculum size (B*C) and between atrazine and trace elements (B*D) appeared to be positive and negative insignificant, on respectively as indicted by *p*-values >0.05. The interaction between the inoculum size and trace elements (C*D) was negative significant. Due to *Ochrobactrum* sp. A7, all factors were found to be positive insignificant at all levels except with the interaction between glucose and the trace elements (A*D), atrazine (B) and trace elements (D*D) which were positive significant at the linear and quadratic levels, respectively while the interaction between glucose and atrazine (A*B), atrazine and trace elements (B*D) and inoculum size and trace elements were negative insignificant.

### Analysis of variance for response surface quadratic model

Analysis of variance (ANOVA) is essential to examine the significance and adequacy of the second-order polynomial model^22^. Prediction of degradation efficiency was determined by the quadratic model, and the significance and interactions of the variables were estimated by ANOVA. The results of ANOVA for the quadratic regression models obtained from CCD used in the optimization of atrazine degradation by *Klebsiella* sp. HA19 and *Ochrobactrum* sp. A7 were in Supplementary Tables S8 and Table S9, respectively. The low *p*-values (0.000) i.e. significant (p< 0.05), revealing that the affirmed quadratic model for atrazine degradation by both isolates was adequate and reliable in defining the actual correlation between response and variables.

### Model validation using residuals

**Fig. 9:**
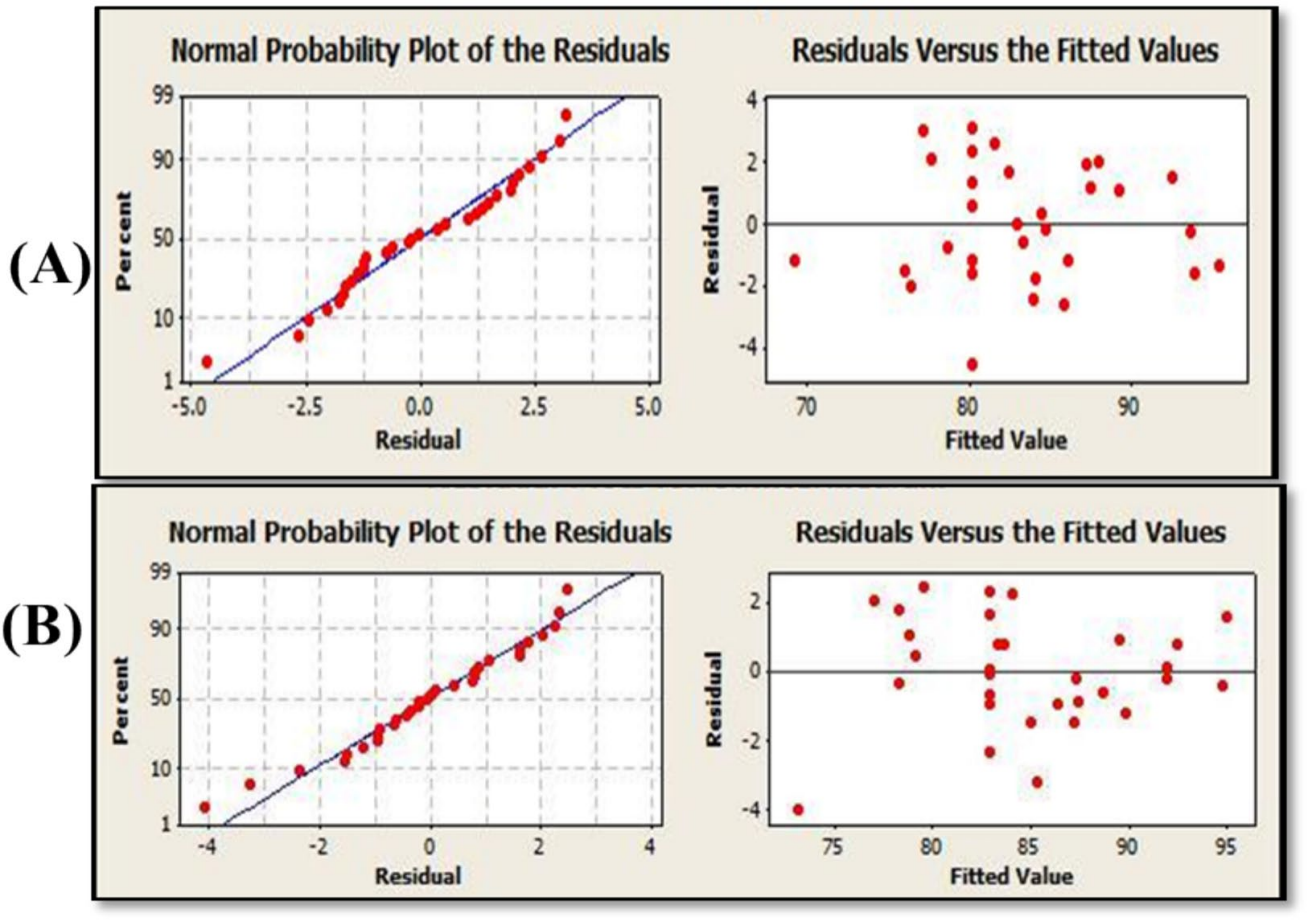
Normal probability plot of residuals against atrazine degradation% and B-Residual distribution against fitted values plot of CCD of (**A**) *Klebsiella* sp. HA19 and (**B**) *Ochrobactrum* sp. A7*.*

Normal probability plots of residual values and prediction success for atrazine degradation percentage by *Klebsiella* sp. HA19 and *Ochrobactrum* sp. A7 are shown in (Fig. 9 A, B**),** respectively. Separation from normality is indicated by the deflection of the straight line. Data points exist as half on the left and half on the right side of the straight line. Both graphs were characterized with approximately accepted straight lines and separating the screen into two portions depending on the data points localized on it. Also the graphs indicated that the data points follow the straight line illustrating that the proposed models are sufficient to show the suitability. According to the graphs, the normal probability plot represents a realistically linear pattern.

### Graphical analysis of the interactive effects of independent variables on atrazine degradation

**Fig. 10:**
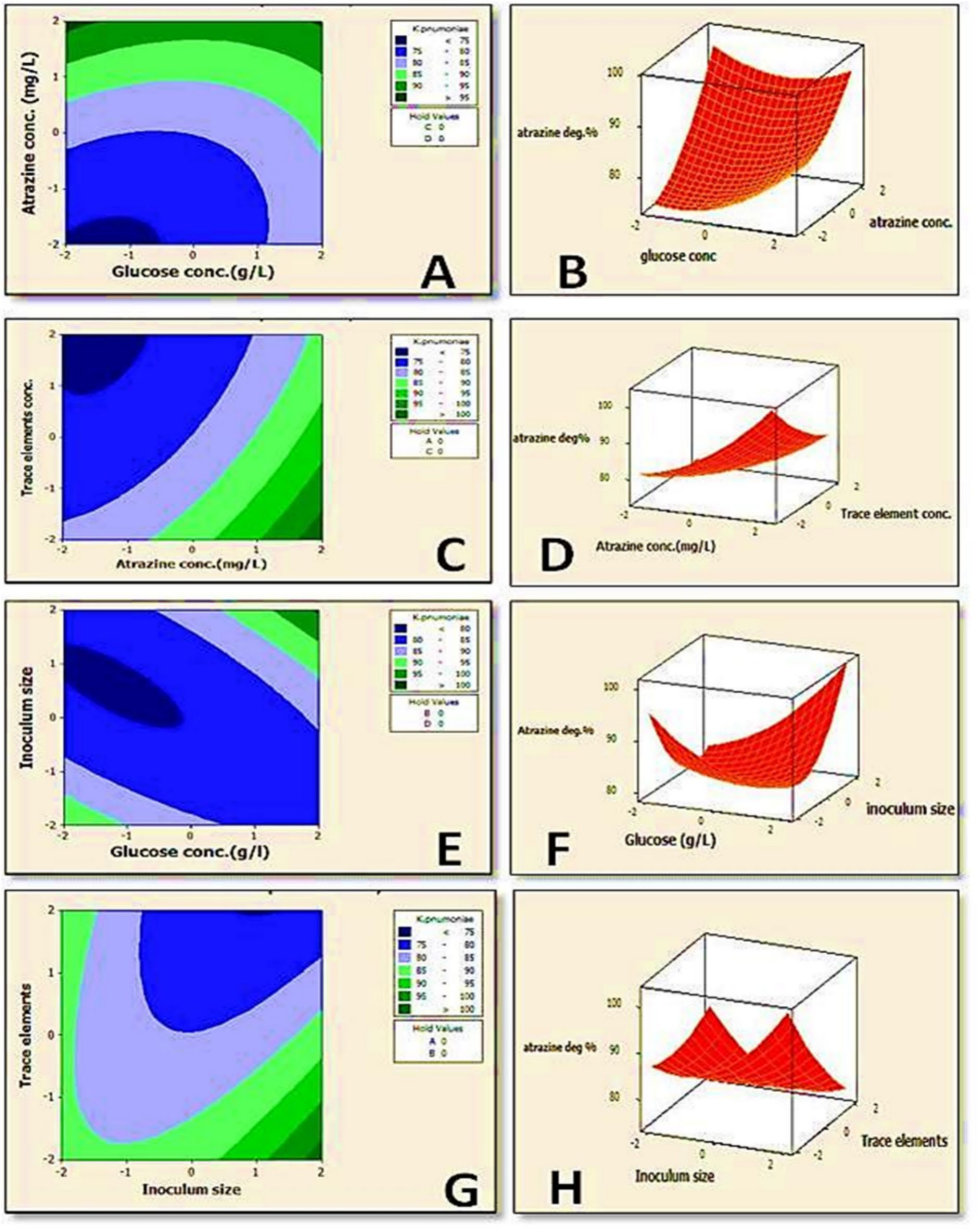
The 3D plots and Contour plots showing the effect of (**A**, **B**). Atrazine and glucose conc. (**C**, **D**). trace elements and Atrazine conc. (**E**, **F**). Inoculum size and glucose conc. (**G**, **H**). Trace elements and inoculum size on atrazine degradation by *Klebsiella* sp. HA19.

**Fig. 11:**
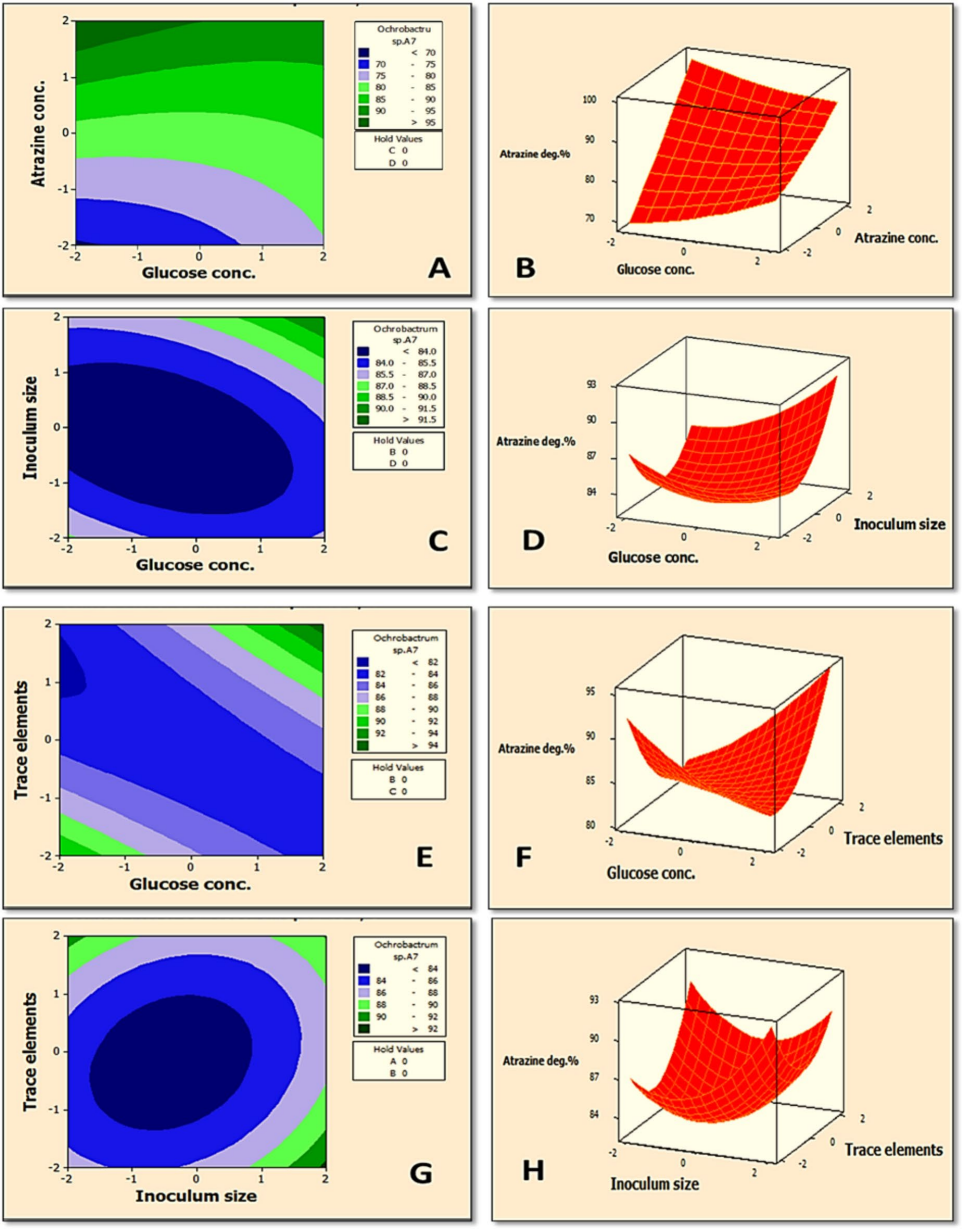
The 3D plots and Contour plots showing the effect of (**A**, **B**). Atrazine conc. and glucose conc. (**C**, **D**). Inoculum size and glucose conc. (**E**, **F**). Trace elements and glucose conc. (**G**, **H**). Trace elements and Inoculum size on atrazine degradation by *Ochrobactrum* sp. A7.

The relationships between the four variables were tested with the help of 2D-contour plots and 3D-surface plots. These graphical representations of contour and response surface were used to demonstrate the effects of two variables on atrazine degradation while the other variables were kept at a fixed level. The interactive effects of the independent variables on atrazine degradation by *Klebsiella* sp. HA19 and *Ochrobactrum* sp. A7 are described in the form of 2D-contour plots (Fig. 10 A, C, E, G and Fig. 11 A, C, E, G) and 3D-response surface plots (Fig. 10 B, D, F, H and Fig. 11 B, D, F, H). Where, optimization factors were denoted as: A = glucose concentration, B = atrazine concentration, C = inoculum size and D = trace elements concentration. The most common feature that characterized the surface plot graphs of both isolates was U-shaped parabola plots.

**Fig. 12:**
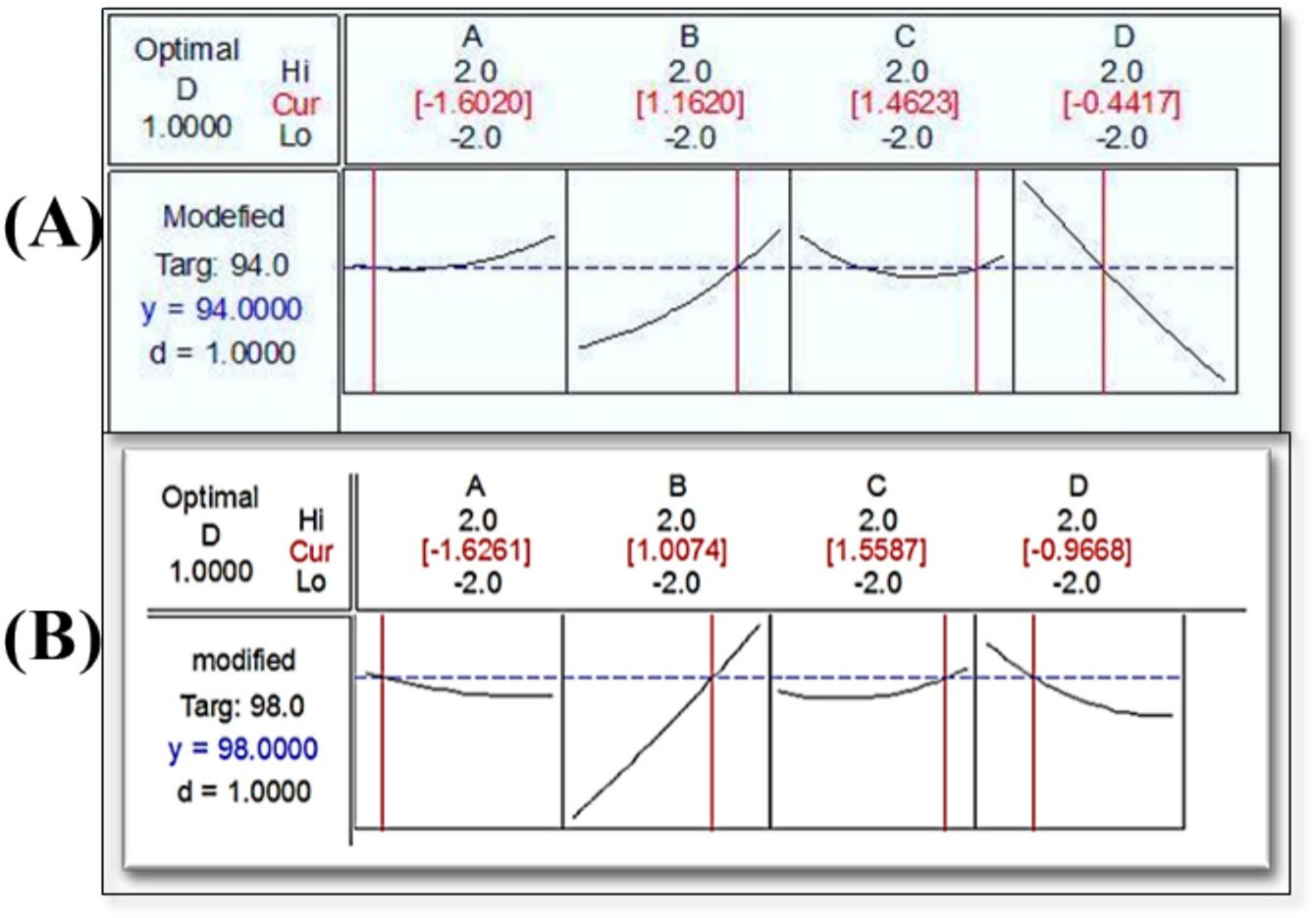
Response optimizer with desirability functions for atrazine degradation (%) with maximum goal of (**A**) *Klebsiella* sp. HA19 (**B**) *Ochrobactrum* sp. A7. (**B1**) *Ochrobactrum* sp. A7 normal cells, (**B2**) Cells of *Ochrobactrum* sp. treated with 100 ppm of atrazine. Arrows and circles indicate the distortion.

Figure 10 represent the analysis graphs of atrazine degradation using *Klebsiella* sp. HA19, but Fig 10 B explained 3D-surface plot describing the effect of glucose concentration and atrazine concentration on the degradation level, results indicated that increasing of glucose concentration to maximum level was correlated with an increase in degradation percentage of atrazine, this was clearly shown as atrazine concentration kept around its centre point (zero- levels).

The interaction between atrazine and trace elements concentrations was illustrated in Fig.10 D. Data showed an elevation of the degradation percentage by increasing atrazine concentration and decreasing of trace elements concentration to minimum levels. Moreover, the 2D-contour plots correlate to the previously mentioned surface plots **(**Fig. 10 A, C**)** displayed almost circular patterns suggesting that the interactions were not statistically significant.

Figure 10E, F clarify the 2D-contour plot and 3D- surface plot as a function of glucose and inoculum size (%) activity at constant values of atrazine and trace elements at their zero levels. It showed that when concentrations of both parameters increased, degradation percentage increased. The elliptical contour plot (Fig.10 E) confirmed the significant synergistic interaction between two factors. The synergistic effect between these two factors meaning that, increasing the rates of glucose and inoculum size has contributed to increase atrazine biodegradation and such results are in agreement with those noted by Eltarhony et al.^21^. It was stated that the shape of the contour plot identifies the nature and degree of the interactions between the variables.

2D-contour plot and 3D- surface plot **(**Fig.10 G, H**)** represent the interactive effect of inoculum size (%) and trace elements concentration on response. It showed that when the concentration of inoculum size (%) increased and trace elements decreased and degradation percentage increased. The elliptical contour plot (Fig.10 G) illustrated the significant antagonistic interaction between the two factors.

The graphical analysis of atrazine degradation for *Ochrobactrum* sp. A7 was shown in Fig. 11. Fig 11 A and B representing the 2D-contour plot and 3D-surface plot demonstrated the interactive effect of glucose and atrazine concentration on the degradation percentage. Degradation percentage increased as atrazine concentration increased and glucose concentration is kept at medium concentration. The circular shaped 2D-contour plot (Fig. 11A) confirmed the insignificant interaction between parameters.

The interaction between glucose concentration and inoculum size demonstrated in Fig. 11 C, D. It showed that the percentage of degradation increased when the values of the two factors increased. 2D-contour plot displayed oval shaped.

A ridged surface plot and a saddle-like 2D-contour plot were observed for the response, reflecting a synergistic effect for the interaction between concentration of glucose and trace elements on atrazine degradation rate, as shown in Fig. 11 E, F. The degradation rate increased with increasing both of the two parameters. A significant interaction was observed between them from the corresponding contour plot.

The correlation of concentration between inoculum size and trace elements was considered to be insignificant as it displayed circular pattern (Fig. 11 G). Maximum degradation percentage could be achieved by increasing the concentration of inoculum size while decreasing trace elements value or vice versa, implying the antagonistic interaction (Fig. 11 H).

### Response surface methodology based optimization

Response optimizer was used to identify the exact optimum combination of the test variable that jointly optimizes response to achieving maximum goals. *Klebsiella* sp. HA19 and *Ochrobactrum* sp. A7 response optimizer figures (Fig.12 A & B) indicated the optimum atrazine degradation conditions and related optimized response factors (independent variables). High and low demonstrated the boundary conditions for degradation conditions and the optimum values are marked in red. *y* axis representing the achieved optimum value while *d* suggests the desirability for each parameter.

Based on these results we can conclude that the optimal condition from this statistical method for obtaining rapid and high rate of atrazine bioremediation are using glucose as a carbon source (glucose) concentration = 0.75gl^-1^, inoculum size= 15% (for both isolates), atrazine concentration= 175ppm, and 150 ppm and trace elements= 4 ml and 3 ml for (*Klebsiella* sp. HA19) and (*Ochrobactrum* sp. A7), respectively.

### Validation of experimental model

The experimental and calculated results were verified by performing confirmation experiments using the optimum conditions predicted by the CCD models. The predicted optimal values of the independent variables of atrazine degradation percentage by *Klebsiella* sp. HA19 and *Ochrobactrum* sp. A7 were shown in Table 1. *Klebsiella* sp. HA19 recorded 84.51% atrazine degradation percentage by application of the predicted optimal conditions compared with 78.6% atrazine degradation percentage by using the basal conditions. The optimal growth condition included 0.75 gl^-1^ carbon source (glucose), 175 ppm atrazine, 15% inoculum size and 4 ml trace elements. In the case of *Ochrobactrum* sp. A7, the optimal growth conditions recorded 86.75% atrazine degradation percentage compared with 79.9% of the basal conditions. Its optimal growth conditions were 0.75 gl^-1^ carbon source (glucose), 150 ppm atrazine, 15% inoculum size and 3 ml trace elements. Based on the obtained results, it can be concluded that the optimized conditions through the selected best CCD model increased the atrazine degradation level by 5.91% and 6.2% with *Klebsiella* sp. HA19 and *Ochrobactrum* sp. A7, respectively, compared to the results of their basal settings.

**Table 1:** Atrazine degradation percentage by *Klebsiella* sp. HA19 and *Ochrobactrum* sp. A7 before and after optimization.

*Klebsiella* sp. HA19				
Independent variables	Values		Response (atrazine degradation %)	
	Basal	Optimum	Basal	Optimum
Glucose conc.(gL-1)	1	0.75	78.6%	84.51%
Atrazine conc.(ppm)	100	175		
Inoculum size (%)	5%	15%		
Trace elements (mlL-1)	5	4		
*Ochrobactrum* sp. A7				
Independent variables	values		Response (atrazine degradation %)	
	basal	optimum	basal	optimum
Glucose conc.(gL-1)	1	0.75	79.9%	86.75%
Atrazine conc.(ppm)	100	150		
Inoculum size (%)	5%	15%		
Trace elements (mlL-1)	5	3		

## Discussion

Civilization and over population are two main reasons that contributed to increase the consumption of pesticides such as atrazine to control the pathogenic microorganisms in order to raise the production rate of crops worldwide particularly in poor countries. Such process of fitting against plant disease using pesticides reflects negatively on the environment by accumulating hazard and toxic chemicals like atrazine in the soil^12^. Serious of environmental problems are bad results of using atrazine due to the half-life of its residents for weeks and months in the soil which dispersed directly to ground water causing hazard effects for human and animals^27^^,^^28^. Consequently, it is urgently to bioremediate this pollutant using safety and friendly way called bioremediation. To achieve this target, a large number of bacterial candidates (44) were isolated from three Governorates (El-Behira, El Meoufiya and Alexandria) that cultivated intensively and treated several times with atrazine in the Nile-Delta and coastal region of Egypt for the last two decades (Supplementary Fig. S3). These isolates were screened to test their ability to use atrazine as a main nitrogen source in agar MSM. About 34 isolates were recognized as atrazine degrading bacteria due to grow on solid MSM containing atrazine and confirming their potentiality to assimilate it as nitrogen source in the presumptive test (Fig. 1). Among them two highly efficient isolates for atrazine degradation were elected from the authentication test based on the estimation of yellow color density (Supplementary Fig. S1) indicating for the presence of atrazine reacted with the substrate of P-aminoacetophenone for yielding polymethine dye^29^. This authentication test confirmed the capability of these two isolates to degrade atrazine effectively and completely, and this was shown clearly from the absent of yellow color which indicating for the presence of atrazine in non-treated tubes or treated ones with less efficient atrazine removing strains.

Subsequently, these two isolates were molecularly characterized to classify them till the species level by using sequencing of targeted genes of 16S rRNA (rrs) and phylogenetic analysis. The sequences of rrs gene of both of the two isolates HA19 and A7 gave high similarity of 99.7% with *Klebsiella sp.* and 95.5% with *Ochrobactrum sp.* using the BLAST tool search at the NCBI (Supplementary Table S2). This high identity confirmed by the phylogenetic affiliation (Fig. 2) supporting for their accuracy identification as *Klebsiella* sp*.* for strain HA19 and *Ochrobactrum* sp*.* for strain A7. Numerous strains have been reported as atrazine degraders belonging to various genera such as *Rhodococcus*^30^, *Agrobacterium*^31^, *Pseudaminobacter*^14^, *Nocardioides*^32^, *Pseudomonas*^33^ and *Bacillus*^34^. Similarly, there are other authors^12^ could detect the ability of strains to degrade atrazine belonging to *Klebsiella sp.* and *Ochrobactrum* sp*.* like in our results.

The physiological behavior of the two examined strains HA19 and A7 for defense against presence of atrazine in the growth medium to survive has been evaluated using SEM (Fig. 3). There was a big damage in the cell wall structure noticed with strain A7 of *Ochrobactrum* sp*.* than strain HA19 of *Klebsiella* sp*..*The production of exopolysaccherides or increase the thickness of cell wall of both strains was noticed as a primary adaptive response mechanism to stresses^35,36^**.**

After selecting the high efficient strains HA19 and A7 for atrazine bioremediation, it was necessary to determine the optimal conditions which can be applied to help these strains for rapid biodegradation of this pollutant. Therefore, the capability of the selected two strains to grow in a wide range of pHs and temperature degrees aiming to accelerate the bioremediation of atrazine was examined under laboratory conditions (data not shown). Our findings indicated that the high degradation rate of atrazine by these two strains noticed at pH between 7-9 degrees which consistent with previous results obtained by Zhang *et al*.^13^ who reported that the alkaline conditions contributed to increase the biodegradation of atrazine, and with the idea stated that atrazine is cationic, its sorption to humic matter and clay is pH dependent^37^**.** The best temperature degree for both strains was 30ºC, while both of higher or lower temperature than this point was associated with reduction in atrazine bioremoval. Such results are in agreement with those noted by Zhang *et al***.**^13^ who published similar findings with strain TT3 of *Citricoccus sp*.

Due to use atrazine as a main nitrogen source by our strains HA19 and A7, it was urgently for searching to find a better carbon source to promote the growth for these strains with an ultimately goal to reduce the required time for completely atrazine bioremoval. Nine different carbon sources (glucose, starch, xylose, sodium citrate, mannitol, lactose, beef, malt extract and molasses) were examined to test their supportive effect on the degradation of atrazine by the two important strains HA19 and A7 (Data not shown). Glucose was reported to be the most important carbon source with the two atrazine degraders as the degradation rate reached to 78.2% and 72.6% on respectively. The promotion effect of glucose on the degradation rate of atrazine by the examined strains was also noted previously by Dehghani *et al*.^38^**.**

The privilege of the previous studies by different authors on atrazine bioremediation by varied microorganisms is to explain three famous pathways that microbes can follow to safety breakdown this hazard compound (Supplementary Fig. S4). Commonly, microorganisms degrade atrazine through water hydrolysis via chlorohydrolase enzyme (*atzA/trzN*) to produce 2-hydroxy atrazine, or dealkylation to produce de-isopropylatrazine (*atrA/thcB*) or genes (*atrA/thcB*) coding for mono-oxygenases enzyme to produce deethylatrazine^39,40^. All the three known pathways of atrazine degradation should generate cyanuric acid as an intermediate which will be converted to biuret by *atzE* gene^39,40^. We successfully amplified the genes *atzA*, *atzC* and *atzD* with both of the two tested strains, but the band of amplified fragment of *atzB* was noticed faintly only with strain A7 of *Ochrobactrum* sp. (Fig. 4). The weak band of *atzB* gene was also noted by^27^ who explained this due to the alkaylamino moieties structure of the herbicide. In contrary to our results Macias-Flores *et al*.^35^ failed to amplify *atzD* in strains belonging to *Klebsiella* sp. and *Ochrobactrum* sp. The presence of *atzA* gene in both of two selected strains in our study indicated that they should follow the degradation pathway of chlorohydrolysis generating 2-hydroxyatrazine. Interestingly, our results of HPLC (Fig. 5) confirmed the existence of deethylatrazine beak associated with alkalylation not 2- hydroxyatrazine generated from chlorohydrolysis. Intriguingly, the incongruent between results of detecting deethylatrazine by HPLC with the amplification of *atzA* gene by PCR can be explained by the possibility of acquisition of these strains for two couple of mechanisms (genes) to degrade atrazine one on the chromosome(*atzA*, *atzB*, *atzC*) and the other on the plasmid (thcB/thcC). This explanation can be supported, by the reports by Piutti *et a*l.^41^ who found that strain SP12 of *Nocardioides* sp. had these *atzB*, *atzC* combined with *trzN* genes which demonstrated clearly for using two different pathways at the same time to biodegrade atrazine similar to our results. Different scientific articles^42,43^ suggested that most of Gram-negative bacteria like our strains have a combination of two sets of genes as atzABC with trzD to mineralize atrazine. Zhang *et al.*^13^ noted that strain FH1 of *Klebsiella variicola* had genes of *atzC*, *trzN* and *trzD* which do not function together and indicating they are not clustered on chromosomal DNA. Indeed, other authors noted the possession of atrazine degrading bacterial strains to these double pathways like trzD with atzD ^44^; trzN with atzC^45^; and trzN with atzBC^46^. An alternative pathway for atrazine degradation started by dealkylation reaction through different way of P-450 cytochrome system has been reported in certain strains of *Rhodococcus* species^30^. The hypothesis that strength our explanations too is the one stated that atrazine degrading genes are common to a wide range of bacterial species due to its easy to move among different genera of soil microorganisms through horizontal gene transfer^15^**.** The inability of detecting ammelide or cyunaric acid as intermediates by our strains (Fig 6) is in agreement with similar findings by James and Singh^47^ who elucidated this to rapid consumption of this byproduct by bacteria prohibiting its accumulation to the level that can be detected by HPLC analysis. Both strains of HA19 and A7 selected in this study were able to biodegrade atrazine till producing biuret because they have a copy of *atzD* gene responsible for convert cyunaric acid to the later bioproduct. Taken together, we can assume that both of our two atrazine degrading strains HA19 and A7 contributed to biodegrade this hazard compound via pathway proposed as the following: (Atrazine, deethyl atrazine, deisopropyl deethylatrazine, (2-chloro-4-hydroxy-6-amino-1,3,5 triazine), (2,4 hydroxy-1,3,5 triazine), cyanuric, then biuret) mainly according to the HPLC data. This pathway of degradation via deetylatrazine is common in alkaline soil^13^ like most of the Egyptian cultivated soils where these two strains were isolated from.

In fact, when atrazine used to control any diseases, then its degradation rate should be affected by the agricultural process as addition of other fertilizers and heavy metals in the soil. Addition of five different fertilizers (urea, ammonium sulphate, calcium nitrate, sodium phosphate mono basic or potassium sulphate) were associated with reduction in the degradation rate of atrazine by 73-75% with both strains compared to control (strains used atrazine as nitrogen source), such observation was similar to those one obtained by Yang *et al*.^48^ (data not shown). The effect of heavy metals was confound, as the low levels of the essential ones of them increased the degradation rate of atrazine while the high levels was inhibited the breakdown of atrazine as previously reported by Kaur *et al*.^49^ data not shown**.**

Khatoon and Rai^5^ used the RSM model to optimize the conditions of the growth medium for obtaining the maximum level of atrazine degradation like in our case while Debasmita and Rajasimman^50^ used it to optimize conditions of atrazin biodegradation in a fermentor. Also it was used for the examination of the relationship between inputs (variables) and outputs (responses) to reach the optimum results^18,51^.

R^2^ determines the prediction power of quadratic regression model (the goodness-of-fit of the model) as noted by Abbas *et al*.^18,51,52^, which ranged from 0–100%. The R^2^ and adjusted R^2^ of *Klebsiella* sp. HA19 and *Ochrobactrum* sp. A7 models were (R^2^= 90.4%, Adj R^2^ = 82%) and (R^2^ =91.9%, Adj. R^2^=84.9%), respectively (Supplementary Table 6 and 7). The proximity of R^2^ and adjusted R^2^ values to 100% were refer to the agreement of experimental and predicted responses and supporting the high significance and adequacy of the model^21,53^.

The F-ratio is usually used to demonstrate the variables differentiation in the data with reference to its mean and lack of fit test which describes the variation in the data around the fitted model testified that insignificant lack of fit indicated that it is a good model^21,54^. The significant *F*-value (10.75 and 13.3) and the non-significant lack-of-fit (0.563 and 0.146) values obtained from the ANOVA declared that the models were statistically significant (Supplementary Tables 8 and 9). Significance of *p*-value (p<0.05), high *F*-ratio and insignificance *p*-value of lack-of-fit (p> 0.05) indicated that the RSM model is statistically significant^5,18^.

Normal probability plot is a graphical method (Fig 9) that used to verify the adequacy of the models and also describes the nature of residuals of the models^18^. Our data points followed the straight line illustrating that the proposed models are sufficient to show the suitability.

Elliptical and saddle-shaped contour plots (Figs. 10 and 11) explicated a significant interaction between variables, whereas a circular contour plot exposes an insignificant interaction between variables^20^.

High and low numbers (Fig. 12) demonstrated the boundary conditions for degradation conditions and the optimum values are marked in red. *y* axis represents the achieved optimum value while *d* suggests the desirability for each parameter^18^.

## Material and methods

### Sample collection

Soil samples were collected from 20 cm top surface layer from different Egyptian fields exposed to atrazine in three governorates El-Behira (Kafr El Dawar; Latitude 31.1311 and Longitude 30.1300), Alexandria (Abis Latitude 31.18793 and Longitude 30.0081) and Borg Alarab; Latitude 30.55239 and Longitude 29.3642) and El Monofeya (Shebin El Kom; Latitude 31.00843 and Longitude 30.54760) for many years and kept at 4 ºC until used (Supplementary Fig. S3). Permission to collect soil samples from the different governorates in Egypt was obtained from our GEBRI institute which has full authority to obtain these soils samples for investigation and analysis.

### Chemicals and reagents

Atrazine (97% purity), deethyl atrazine (98% purity), ammelide (98% purity) and cyanuric acid (≥98% purity) were purchased from Sigma Aldrich, USA. Other chemicals used in this study were of analytical grade obtained from Difco, Oxoid, Sigma and Fluka Companies, and those used for HPLC analysis, were of HPLC grade. Atrazine dissolved in methanol, to make stock solution of 100 mg l^-1^ and then stored at 4° C prior to use.

### Bacterial isolation

Ten-grams of fine soil were added to 250 ml Erlenmeyer flask containing 100 ml of 50 mg l^-1^ atrazine in (MSM) minimal salt medium^30^, and incubated aerobically at 30°C at 150 rpm for 7 days. After that, 10 ml from the enriched culture was transported to fresh MSM^30^ containing higher concentration of atrazine (100 mg l^-1^). This process repeated 10 times to obtain a final atrazine concentration of 1000 mg l^-1^. All enrichment cultures were serially diluted, plated on LB ^48^ agar plates containing atrazine (200 mgl^-1^), and incubated at 30°C. A group of selected colonies were repeatedly streaked on LB agar plates to have a pure culture.

### Detection of atrazine degraders using color method

A single colony of each isolate was transferred to 100 ml^-1^ MSM containing 100 mg l^-1^ atrazine to test its biodegradation ability by measuring the yellow color at 470 nm spectrophotometrically by the substrate *p*-aminoacetophenone as explained by Kesari and Geputa 1998^29^. This method depending on the formation of quaternary halide (QH) due to reacting atrazine with pyridine, and QH takes hydroxyl group from alkaline condition that breaks it and forming glutaconic dialdehyde which detected by the substrate *p*-aminoacetophenone (PMP) by producing the yellow orange of polymethine dye, then the high color of yellow dye indicating for the presence and concentration of atrazine, while the absence of this color is an evident for biodegradation of atrazine by our strains. The reaction was done as the following; atrazine was extracted from culture supernatant by dichloromethane. The extracts concentrated and left to be dry on air then dissolved with methanol HPLC-grade. 2.5 ml of atrazine extract put in a graduated tube and mixed with 0.2 ml of pyridine reagent. Tubes placed in boiling water for 15 min and cooled at room temperature. One ml of 2M NaOH and 2 ml of p-aminoacetophenone solutions were added and mixed well then left 5 min for color development and the absorbance measured at 470 nm on spectrophotometer model 752PRO.

### Biochemical tests of atrazine degrading isolates

Isolates were biochemically characterized using the kit of Microbate 12E (OXOID, England) which used to identify *Enterobacteriaceae* and other Gram negative bacteria according to the manufacture instructions. Detail’s about the method is described by Tamba et al.^55^.

### Preservation of atrazine degrading strains

Strains that proved their ability to biodegrade atrazine were grown on MSM broth amended with atrazine and after 72h of growth, aliquots of the growth distributed on the surface of MSM agar to obtain single colonies then these colonies were scraped by sterile cotton tap and mixed with 50% glycerol with MSM broth and kept at -80^ο^ C.

### Estimation of atrazine biodegradation percent

A serial dilution of atrazine was made to obtain different concentrations from 10 to 100µg ml^-1^ of the standard atrazine mentioned above to create a standard relationship curve between atrazine concentration and its absorbance at 470 nm wave length. The residual concentration of atrazine after treated with atrazine biodegrading strains was evaluated from the equation Y= 0.0123xX-o.o731. Where Y, is the absorbance, and X is the atrazine concentration. Then atrazine biodegradation percent was accounted from the equation obtained by Yang *et al*.^43^ as the following:

X= (Cck-CX) ÷ CcKx100, where X is the percent of atrazine degradation, CX is the final concentration of atrazine and Cck is the original concentration of atrazine^48^.

### DNA extraction

Genomic DNA from each bacterial isolate was extracted from 1 ml bacterial culture grown overnight after the cell lysis with SDS** (**Sodium dodecyl sulfate**)** and it collected by CTAB (Cetyltrymethyl-ammoniumbromide), then DNA precipitated by isopropanol after purification with Chloroform/Isoamyl alcohol according to the standard method described by Ausubel *et al*.^56^.

### 16S rRNA amplification, sequencing and phylogenetic analysis

Amplification of 16S rRNA gene was done using the universal primers of R5 and R6^57^ and the template DNA of selected strains using (3 Prime Thermal cycler, TECHNE, UK). One µl of DNA, 5 µl of the 10x polymerase reaction buffer, 4 µl of 25 mM MgCl_2_, 2 µl of each primer, 4 µl of dNTPs (2.5 mM), 1 µl of 1U µl^-1^Taq DNA polymerase. Thermo-cycler conditions of PCR was as the following: an initial denaturation temperature for 5 min at 95ºC, 25 cycles for 1 min at 95ºC, annealing for 1 min at 52ºC, extension for 2 min at 72ºC and final extension time (7 min at 72ºC). The product of amplified 16S rRNA (10 µl plus loading dye) was checked by running on 1% agarose gel in TBE buffer using horizontal electrophoresis unit. The amplified band detected under UV light after staining the gel with 0.5 µg ml^-1^ of ethidium bromide. The product of 16S rRNA was cleaned using QIAquick clean kit from Qiagen Company. The purified yield was sequenced using Perkin Elmer 377 DNA automated sequencer and Dye Deoxy Terminator Cycle (Perkin Elmer, Foster City, CA) with the forward and reverse primers that used before for amplification as described by Van Berkum *et al*.^58^. Sequences of the two captured strains were deposited in the Gene Bank under accession numbers OL815019 and OL815020 Phylogenetic trees were conducted using MEGA version 6.0. The tree was constructed using the neighbour-joining method, and bootstrapping was performed for 1000 replicates. After identification based on molecular analysis, the high efficient strains HA19 and A7 or atrazine degraders were identified.

### Detection of atrazine degrading genes

Amplification of four atrazine degrading genes, including *atzA*, *atzB*, *atzC*, and *atzD*, was performed using primers published by Van Berkum and Fuhrmann^59^ and Zhang et al.^13^. The reaction mix and condition of PCR cycles was similar to those ones mentioned above for amplifying a part of ribosomal RNA, except the annealing temperature that varied from gene to another and noted in the reports obtained by Van Berkum and Fuhrmann^59^ and Zhang et al.^13^. The yield of PCR separated on 1.5% agarose gels and detected under UV light after staining with ethidium bromide.

### Optimization of medium growth conditions

To select the best conditions for growing atrazine degraders five different pH (3, 5, 7, 9, 11) and five temperature degrees 15, 25, 30, 35, 42°C were applied in the growth medium of agar MSM to know which one of them will accelerate the growth of bacterial strains.

### Scanning electron microscopy (SEM) to assess cellular changes of strains

Atrazine-induced cellular damage and distortion in the cell walls of the two strains HA19 *K. pneumoniae* and A7 *O. intermedium* strains were observed under SEM by growing the bacterial in LB medium amended with 100 µg ml^−1^ atrazine, and incubated at 30 °C for seven days. Atrazine-free culture medium inoculated with bacterial cells used as control. After incubation, bacterial cells were centrifuged for 10 min at 10,000 rpm and pellets washed twice by 1X phosphate buffer saline (PBS) and pre-fixed overnight at 4 °C with 2.5% glutaraldehyde. The cell pellets were washed again two times with PBS and centrifuged for 5 min at 10,000 rpm. After washing, the fixed specimens were dehydrated in a graded series of ethanol for 5 min. Then, cell pellets were centrifuged and re-suspended in PBS. Five μl of bacterial suspension smeared on cover slip and dried. The specimens were mounted on aluminium stub, coated with gold and analysed under the SEM model (JEOL JSM6360LA Japan) to see the morphological changes in bacterial cell walls^13^.

### HPLC tool to analyse metabolites of atrazine degradation

After extracting atrazine from culture supernatant, aqueous samples were filtrated through a 0.22 μm nylon filter. The concentration of atrazine and yield of its metabolites from each sample were measured by HPLC (Agilent 1100). For atrazine and deethylatrazine detection, the HPLC was equipped with a variable wavelength UV detector set to 225 nm and fitted with a reverse-phase C18 column (4.6 × 250 mm, 5 μm) with a flow rate of 1.0 ml min ^-1^ (methanol/water = 70/30, v/v) and a column temperature of 40 °C. To detect the yield of cyanuric acid and ammelide, HPLC was equipped with a variable UV detector set at a wavelength of 215 nm and fitted with a Thermo NH_2_ column (4.6 × 250 mm, 5 μm), with a flow rate of 1.0 ml min^-1^ (methanol/0.1% NH_3_·H_2_O = 90/10, v/v) and a column temperature of 30 °C. All injection runs were performed using a 10μl of each sample^13^.

### Heavy metal stock solutions

Analytical grade salts of CuSO_4_, Co(NO_3_)_2_, CrCl_3_.6H_2_O, NiCl_2_.6H_2_O, CaCl_2_.H_2_O, ZnCl_2_, Fe(NO_3_)_2_, CdCl_2_, (CH_3_COO)_2_Pb (lead acetate), AgNO_3_ and HgSO_4_ were used to prepare 0.125 M stock solutions for each one^49^.

### RSM experimental design

To optimize the concentration of four variables CCD model of (RSM) were used to predict their concentrations and give the highest atrazine degradation rate^5^. These factors were (glucose, atrazine, trace elements concentrations, and inoculum size). Each one was studied with five different values as explained in (Table 2).

**Table 2:** Coded levels with experimental values of some factors for CCD variables.

Variable	Unit	Coded level/experimental values				
		-2	-1	0	1	2
Glucose (X1)	gl^-1^	0.5	1	2.5	5	10
Atrazine (X2)	µg/ml	25	50	100	150	200
Trace elements (X3)	ml/L	1	3	5	10	20
Inoculum size (X4)	ml	1%	3%	5%	10%	20%

A 16-factorial design with eight axial points and seven replicates at the centre point with a total of 31 trials was run as indicated in (Supplementary Table S1). Atrazine biodegradation rate was taken as response (Y) and a multiple regression analysis of the data was done to obtain an experimental model that relates the response measured to the independent variables. At each experiment, atrazine was extracted and determined after seven days of incubation time. Each experiment was performed in triplicate and the average calculated for subsequent statistical analysis.

For the statistical calculation, the relationship between the coded and the actual values is described by the following equation:

3 $$Xi\hspace{0.17em}=\hspace{0.17em}Ui-Ui0/\Delta Ui$$

Where *Xi* is the coded value of the *i*th variable, *Ui* is the actual value of the *i*th variable, Ui0 is the actual value of the *i*th variable at the centre point, and ΔUi is the step change of variable. The response variable (atrazine degradation %) suitable to a quadratic equation for the variables was as Eq.2:

4 $$\begin{aligned} Y\hspace{0.17em}&=\hspace{0.17em}\beta 0\hspace{0.17em}+\hspace{0.17em}\beta 1X1\hspace{0.17em}+\hspace{0.17em}\beta 2X2\hspace{0.17em}+\hspace{0.17em}\beta 3X3\hspace{0.17em}\\&+\hspace{0.17em}\beta 11X11\hspace{0.17em}+\hspace{0.17em}\beta 22X22\hspace{0.17em}+\hspace{0.17em}\beta 33X33\hspace{0.17em}\\&+\hspace{0.17em}\beta 12X1X2\hspace{0.17em}+\hspace{0.17em}\beta 13X1X3\hspace{0.17em}+\hspace{0.17em}\beta 23X2X3\end{aligned}$$

Where: *Y* is the predicted response; X_1_, X_2_, X_3_ are input variables which influence the response variable Y*; β*_0_, intercept (term constant); *β*_1_, *β*_2_ and *β*_3_ linear coefficients; *β*_11_, *β*_22_ and *β*_33_, squared or quadratic coefficients *β*_12_, *β*_13_, and *β*_23_ interaction coefficients^5^.

### Residual atrazine extraction and estimation

After incubation, atrazine residue extracted from sample by liquid-extraction method. Five ml aliquots were taken from each broth culture and centrifuged to obtain cell-free supernatant, and then 5 ml of dichloromethane was added to the solution and shaken vigorously for 3 min. The whole content was allowed to stand-quiet, for separating the water and dichloromethane-layer. This process was repeated two times with the aqueous phase by addition 5 ml dichloromethane each time. The combined of organic extracts were concentrated and left to dry in the air, and then, the residue was dissolved in 1ml HPLC-grade methanol, then the residual atrazine was measured by HPLC (Agilent 1100) equipped with a variable wavelength UV detector set to 225 nm and fitted with a reverse-phase C18 column (4.6 × 250 mm, 5 μm) with a flow rate of 1.0 ml/min (methanol/water = 70/30, v/v) and a column temperature of 40°C^13^.

The initial and final concentration of atrazine measured, and the percentage of atrazine biodegradation was calculated. The percentage of atrazine degraded can be calculated as described by Yang *et al*.^48^ from Eq.3**:**

5 $$X\hspace{0.17em}=\hspace{0.17em}(C_{CK}\hspace{0.17em}-\hspace{0.17em}C_{X})/C_{CK}\hspace{0.17em}\times \hspace{0.17em}100{\%}$$

Where X is the degradation percentage of atrazine, C_X_ is the final concentration of atrazine (mgl^-1^), and C_CK_ is the original concentration of atrazine (mgl^-1^).

### Statistical analysis

Minitab 15.0 (Minitab Inc., Pennsylvania, USA) used to develop the experimental designs (matrices), subsequent regression analysis of the experimental data of CCD, perform analysis of variance (ANOVA), 3D surface plots, 2D contour plots and optimizer. The quality of the polynomial model equation was judged statistically by the coefficient of determination R2, and its statistical significance was determined by F-test^19,60,61^.

## Conclusion and plane in the future

About 77% of the tested isolates were reported to degrade atrazine in MSAM, indicating that the genes responsible for atrazine bioremediation are dominant in a wide range of microorganisms. Strains HA19 and A7 were selected as highly effective strains for bioremoving atrazine with efficiency rate of 81% and 81.3% on respectively depending on both presumptive or authentication tests. These two strains HA19 and A7 were identified as *Klebsiella* sp*.* and *Ochrobactrum* sp*.* using molecular toots as sequencing and phylogenetic analysis of 16S rRNA. Optimization of growth medium conditions to accelerate the efficiency of these strains to bioremediate this hazard chemical indicated that supplementing the growth medium with 0.75 g glucose per liter supported the heavy growth of strains, the optimum pH was around 7-9, and temperature is 30 ºC. The suggested degradation pathway of atrazine is the one started by an intermediate deethylatrazine based on HPLC analysis of metabolites. Presence of atrazine degrading genes like *atzA*, *atzB*, *atzC* and *atzD* and high efficiency of these two strains proved their potentialities to be used to eliminate or detoxify atrazine in contaminated soil sites.

The use of statistical RSM model based on CCD was used to predict the optimum concentrations of four important variables glucose, atrazine, trace elements and inoculum size simultaneously on atrazine biodegradation aiming to safety and quicker bioremovel of this pollutant from the environment. Such model confirmed its usefulness as powerful tool, in selecting optimum degradation conditions that were glucose concentration = 0.75gl^-1^, inoculum size= 15% (for both strains), atrazine concentration 175ppm, and 150 ppm and trace elements= 4 ml and 3 ml for *Klebsiella* sp. HA19 and *Ochrobactrum* sp. A7, respectively. It is also contributed to raise the biodegradation rate of atrazine by about 5% over than the one variable method and of course reduced the time of bioremediation.

We will focus in the future to analyze both of atrazine degrading gens on plasmid and chromosomal DNA to verifying the expected mechanisms that these strains acquire to biodegrade atrazine and the possibility of transferring these genes to new recipients strains like *Rhizobium* to use them as nitrogen fixers and atrazine bioremoval simultaneously. Also we will direct our efforts to study the use of these two atrazine degraders (HA19 and A7) in a soil polluted with atrazine and examine their validity to give soil free from atrazine contamination after treating the polluted sites with them and test the suitability of this soil to cultivate with non-edible plants like acacia seedlings.

## Supplementary Information

Supplementary Information.

## Data Availability

The datasets generated and/or analyzed during the current study are available in the NCBI website, under accession no OL815019.1 and OL815020.1 (https://www.ncbi.nlm.nih.gov/nuccore).
