# Supplementary material for: Optimized atrazine biodegradation in Egyptian soils via native bacterial isolates and molecular insights using different statistical methods
Source: Sci Rep. 2026 Jun 16;16:18710. doi: 10.1038/s41598-026-55623-5 (PMC13272960; doi:10.1038/s41598-026-55623-5)
Supplement: Supplementary file 1 — Supplementary Information. [file 41598_2026_55623_MOESM1_ESM.pdf]

Supplementary information

Figure legends

Supplementary Fig. S1. Colorimetric method for detection of atrazine bioremediation through producing polymethine dye (yellow color) as a result of interaction between atrazine and P-aminoacetophenone.

Supplementary Fig. S2. Growth curve and atrazine degradation by (A) *Klebseilla sp.* HA19 and (B) *Ochrobactrum sp.* A7.

Supplementary Fig. S3. Number of bacterial cultures isolated from soil samples collected from different Egyptian fields polluted with atrazine.

Supplementary Fig. S4. The three famous pathways of atrazine
[35](2009 ,.biodegradation by different kinds of microorganisms (Govantes et al

.

Table legends

Supplementary Table S1. Morphological and biochemical characteristics of HA19 and A7.

Supplementary Table S2. Similarity Percentages of our two strains compared with identified strains from the gene bank.

Supplementary Table S3. Matrix of CCD of response surface methodology for optimization of atrazine biodegradation.

Supplementary Table S4. Experimental & predicted results of atrazine degradation from Five–Level CCD of four variables by *Klebsiella* sp. HA19.

Supplementary Table S5. Experimental & predicted results of atrazine degradation from five–level CCD of four variables by *Ochrobactrum* sp. A7.

Supplementary Table S6. Estimated Regression Coefficients for second order polynomial model of atrazine degradation by *Klebsiella* sp. HA19**.**

Supplemenary Table S7. Estimated Regression Coefficients for second order polynomial model of atrazine degradation *Ochrobactrum* sp. A7

Supplementary Table S8. ANOVA for the quadratic polynomial model for atrazine degradation by *Klebsiella* sp. HA19**.**

Supplementary Table S9. ANOVA for the quadratic polynomial model for atrazine degradation by *Ochrobactrum* sp. A7

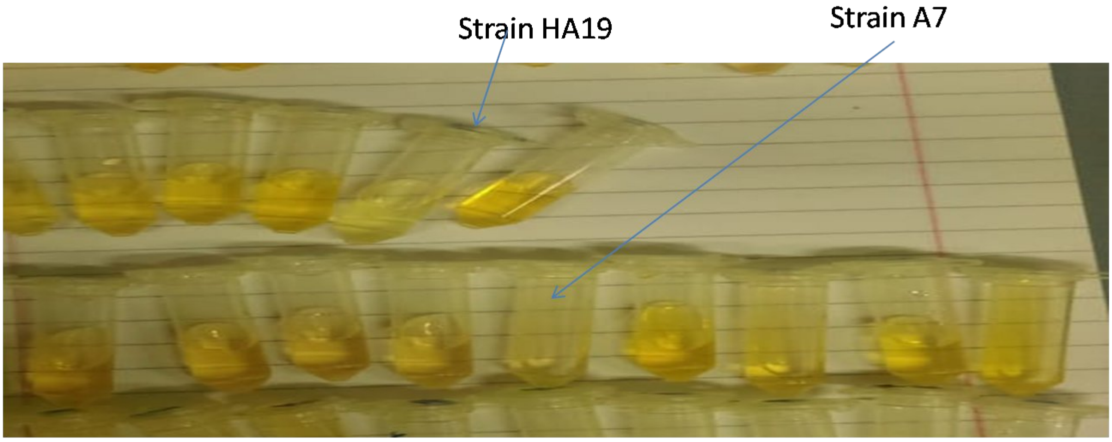

Supplementary Fig. S1.

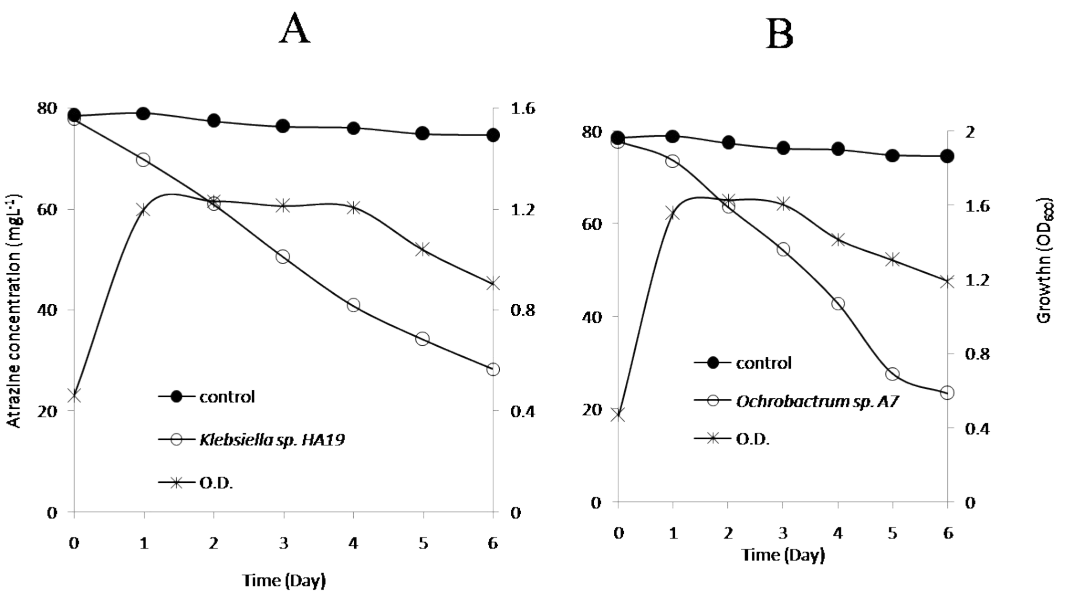

Supplementary Fig. S2

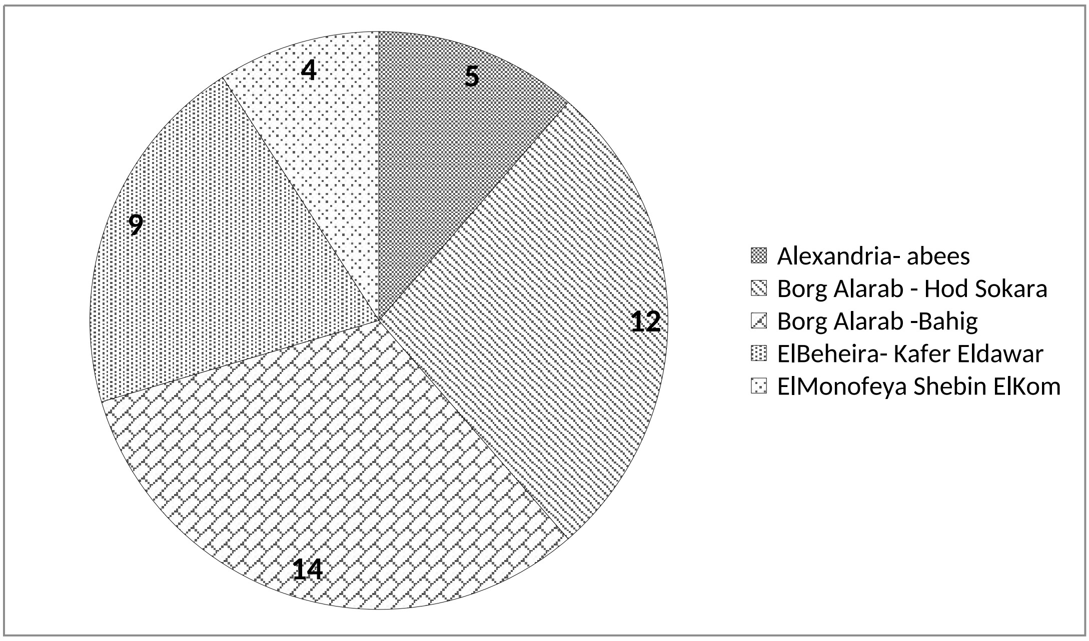

Supplementary Fig. S3

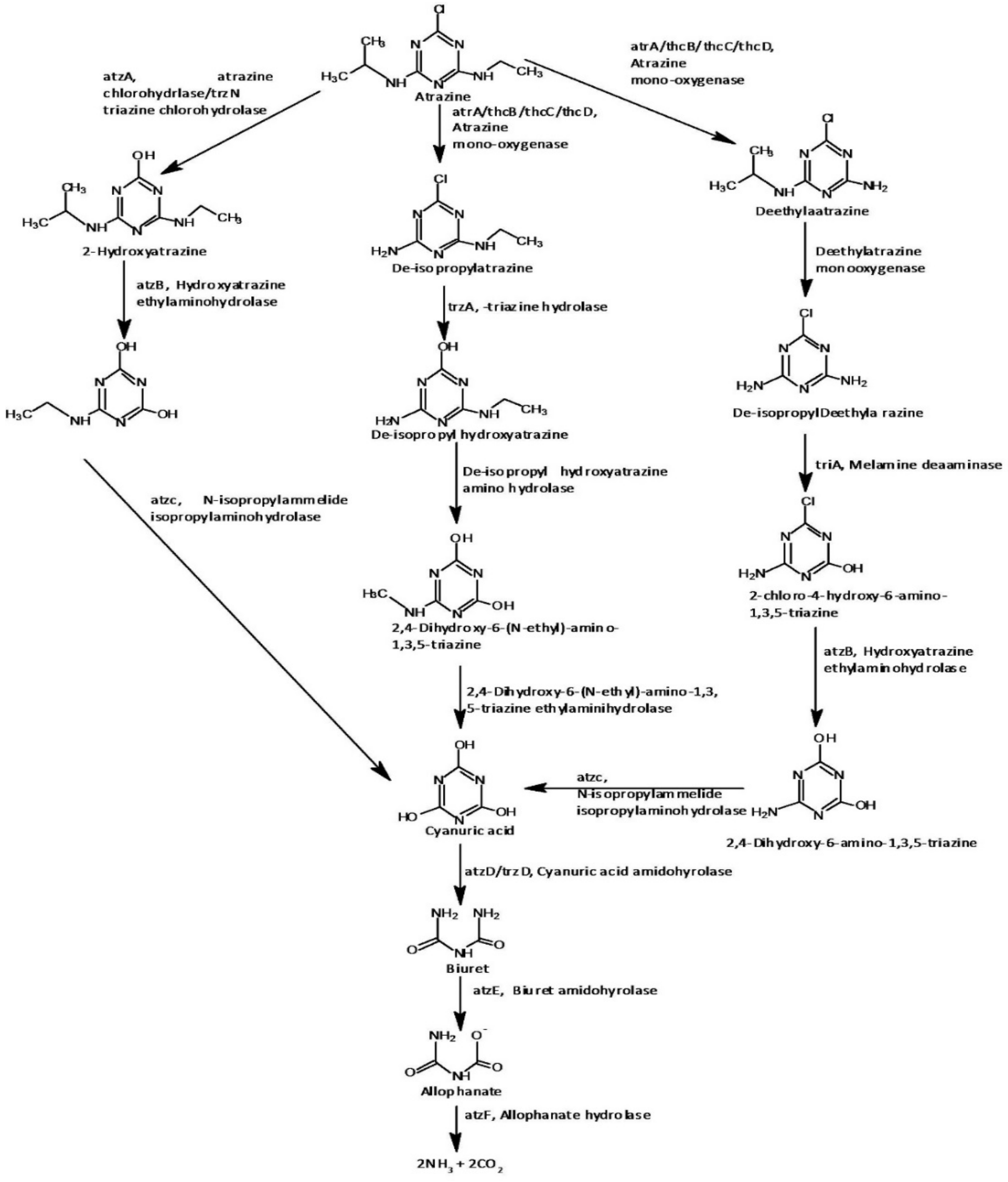

Supplementary Fig. S4

Supplementary Table S1. Morphological and biochemical characteristics of HA19 and A7

Criteria	HA19	A7
**Morphological characteristics**		
Cell shape	Short rods	Rods
Gram stain	Negative	Negative
Motility	Non motile	Non-motile
Endospore	Non-spore forming	Non-spore forming
Pigment	Colorless	Colorless
**Biochemical tests**		
Lactose fermentation	Positive	Negative
Lysine	Positive	Positive
Ornithine	Positive	Positive
H_2S	Negative	Negative
Glucose	Positive	Positive
Mannitol	Positive	Positive
Xylose	Negative	Positive
ONPG	Positive	Positive
Indole	Negative	Negative
Urease	Positive	Positive
V.P.	Positive	Positive
Crate	Positive	Positive
TDA	Positive	Positive
Nitrate reduction	Positive or negative	Positive or negative

Supplementary Table S2. Similarity percentages of our two strains compared with identified strains from the gene bank

Description	Scientific name	Query cover	Identity %	Sequence length	Accession number
**Isolate HA19**					
Klebsiella sp. HA19	*Klebsiella* sp.	100%	100%	1201	OL815019.1
Klebsiella pneumoniae sd-2	*Klebsiella p.*	95%	99.39%	1383	KU353687.1
**Isolate A7**					
Ochrobactrum sp. A7	*Ochrobactrum* sp.	100%	100%	1450	OL815020.1
Ochrobactrum sp. KT48	*Ochrobactrum* sp.	99%	99%	1358	KJ734021.1

Supplementary Table S3. Matrix of CCD of response surface methodology for optimization of atrazine biodegradation

Run Order	Glucose	Atrazine conc.	Inoculum size	Trace elements
1	0	0	0	0
2	-1	-1	-1	-1
3	0	0	2	0
4	0	0	-2	0
5	2	0	0	0
6	-1	1	-1	1
7	-1	1	-1	-1
8	0	0	0	0
9	1	1	1	-1
10	-1	-1	1	-1
11	1	-1	1	-1
12	1	-1	-1	-1
13	0	0	0	2
14	1	1	-1	1
15	1	-1	1	1
16	0	0	0	0
17	0	0	0	0
18	0	-2	0	0
19	1	-1	-1	1
20	0	2	0	0
21	-1	-1	1	1
22	0	0	0	0
23	0	0	0	0
24	1	1	-1	-1
25	-1	1	1	-1
26	1	1	1	1
27	-2	0	0	0
28	-1	-1	-1	1
29	0	0	0	0
30	-1	1	1	1
31	0	0	0	-2

Supplementary Table S4. Experimental & predicted results of atrazine degradation from Five–Level CCD of four variables by *Klebsiella* sp. HA19

Run order	Run type	A	B	C	D	Experimental Atrazine degradation%	Predicted Atrazine degradation%	Standardized Residual
1	central	0	0	0	0	83.3	80.129	1.3
2	factorial	-1	-1	-1	-1	81.5	83.942	-1.44
3	axial	0	0	2	0	88.7	87.479	0.72
4	axial	0	0	-2	0	84.9	86.129	-0.72
5	axial	2	0	0	0	83.2	85.863	-1.57
6	factorial	-1	1	-1	1	82.4	84.158	-1.03
7	factorial	-1	1	-1	-1	93.5	93.763	-0.15
8	central	0	0	0	0	75.5	80.129	-1.9
9	factorial	1	1	1	-1	92.4	94.046	-0.97
10	factorial	-1	-1	1	-1	82.7	83.279	-0.34
11	factorial	1	-1	1	-1	84.9	84.525	0.22
12	factorial	1	-1	-1	-1	79.8	77.663	1.26
13	axial	0	0	0	2	74.4	76.413	-1.18
14	factorial	1	1	-1	1	89.2	87.229	1.16
15	factorial	1	-1	1	1	84.1	82.446	0.97
16	central	0	0	0	0	80.7	80.129	0.23
17	central	0	0	0	0	82.5	80.129	0.97
18	axial	0	-2	0	0	74.5	75.996	-0.88
19	factorial	1	-1	-1	1	82.9	82.908	0
20	axial	0	2	0	0	94.1	92.613	0.88
21	factorial	-1	-1	1	1	67.9	69.075	-0.69
22	central	0	0	0	0	78.5	80.129	-0.67
23	central	0	0	0	0	81.5	80.129	0.56
24	factorial	1	1	-1	-1	84.5	84.708	-0.12
25	factorial	-1	1	1	-1	94.2	95.575	-0.81
26	factorial	1	1	1	1	90.3	89.242	0.62
27	axial	-2	0	0	0	84.2	81.546	1.56
28	factorial	-1	-1	-1	1	80.1	77.063	1.79
29	central	0	0	0	0	78.9	80.129	-0.5
30	factorial	-1	1	1	1	77.9	78.646	-0.44
31	axial	0	0	0	-2	90.1	88.096	1.18

Supplementary Table S5. Experimental & predicted results of atrazine degradation from five–level CCD of four variables by *Ochrobactrum* sp. A7

Run order	Run type	A	B	C	D	Experimental atrazine degradation %	Predicted atrazine degradation %	Standardized Residual
**1**	central	0	0	0	0	82.2	82.857	-0.33
**2**	factorial	-1	-1	-1	-1	79.9	78.833	0.76
**3**	axial	0	0	2	0	86.5	87.417	-0.65
**4**	axial	0	0	-2	0	83.5	85.017	-1.08
**5**	axial	2	0	0	0	82.1	85.35	-2.31 R
**6**	factorial	-1	1	-1	1	88.1	88.717	-0.44
**7**	factorial	-1	1	-1	-1	91.8	92	-0.14
**8**	central	0	0	0	0	84.5	82.857	0.81
**9**	factorial	1	1	1	-1	92.1	91.983	0.08
**10**	factorial	-1	-1	1	-1	77.9	78.233	-0.24
**11**	factorial	1	-1	1	-1	82	79.517	1.77
**12**	factorial	1	-1	-1	-1	80.1	78.317	1.27
**13**	axial	0	0	0	2	85.7	87.183	-1.06
**14**	factorial	1	1	-1	1	90.4	89.5	0.64
**15**	factorial	1	-1	1	1	84.4	83.633	0.55
**16**	central	0	0	0	0	82.9	82.857	0.02
**17**	central	0	0	0	0	82.8	82.857	-0.03
**18**	axial	0	-2	0	0	69	73.05	-2.88 R
**19**	factorial	1	-1	-1	1	86.3	84.033	1.61
**20**	axial	0	2	0	0	96.7	95.083	1.15
**21**	factorial	-1	-1	1	1	79	76.95	1.46
**22**	central	0	0	0	0	80.5	82.857	-1.17
**23**	central	0	0	0	0	81.9	82.857	-0.47
**24**	factorial	1	1	-1	-1	87.2	87.383	-0.13
**25**	factorial	-1	1	1	-1	94.4	94.8	-0.28
**26**	factorial	1	1	1	1	93.3	92.5	0.57
**27**	axial	-2	0	0	0	84.1	83.283	0.58
**28**	factorial	-1	-1	-1	1	79.6	79.15	0.32
**29**	central	0	0	0	0	85.2	82.857	1.16
**30**	factorial	-1	1	1	1	88.7	89.917	-0.87
**31**	axial	0	0	0	-2	85.4	86.35	-0.68

R denotes an observation with a large, standardized residual.

Supplementary Table S6. Estimated Regression Coefficients for second order polynomial model of atrazine degradation by *Klebsiella* sp. HA19

Term	Coef	SE Coef	T	P
Constant	80.1286	0.9948	80.547	0
Glucose	1.0792	0.5373	2.009	0.062 NS
atrazine	4.1542	0.5373	7.732	0 *
Inoculum size	0.3375	0.5373	0.628	0.539 NS
Trace elements	-2.9208	0.5373	-5.437	0 *
$(Glucose)^2$ A*A	0.8939	0.4922	1.816	0.088 NS
$(Atrazine)^2$ B*B	1.0439	0.4922	2.121	0.05 NS
$(Inoculum\ size)^2$ C*C	1.6689	0.4922	3.391	0.004 *
$(Trace\ elements)^2$ D*D	0.5314	0.4922	1.08	0.296 NS
Glucose *atrazine A*B	-0.6938	0.658	-1.054	0.307 NS
Glucose* inoculum size A*C	1.8812	0.658	2.859	0.011 *
Glucose*trace elements A*D	3.0312	0.658	4.607	0 *
Atrazine*inoculum size B*C	0.6187	0.658	0.94	0.361 NS
Atrazine* trace elements B*D	-0.6812	0.658	-1.035	0.316 NS
Inoculum size*trace elements C*D	-1.8313	0.658	-2.783	0.013 *
**R^2 = 90.4%** **R^2 (adj) = 82.0%**<br>*** (significant <0.05) , NS (non significant >0.05)**				

Supplementary Table S7. Estimated Regression Coefficients for second order polynomial model of atrazine degradation *Ochrobactrum* sp. A7

**Term**	**Coef**	**SE Coef**	**T**	**P**
Constant	82.8571	0.8231	100.671	0
Glucose	0.5167	0.4445	1.162	0.262 NS
Atrazine	5.5083	0.4445	12.392	0 *
Inoculum size	0.6	0.4445	1.35	0.196 NS
Trace elelments	0.2083	0.4445	0.469	0.646 NS
$(\text{Glucose})^2$ A*A	0.3649	0.4072	0.896	0.384 NS
$(\text{Atrazine})^2$ B*B	0.3024	0.4072	0.743	0.469 NS
$(\text{inoculum size})^2$ C*C	0.8399	0.4072	2.062	0.056 NS
$(\text{trace elements})^2$ D*D	0.9774	0.4072	2.4	0.029 *
Glucose*atrazine A*B	-1.025	0.5444	-1.883	0.078 NS
Glucose*inoculum size A*C	0.45	0.5444	0.827	0.421 NS
Glucose*trace elements A*D	1.35	0.5444	2.48	0.025 *
Atrazine* inoculum size B*C	0.85	0.5444	1.561	0.138 NS
Atrazine* trace elements B*D	-0.9	0.5444	-1.653	0.118 NS
Inoculum size* trace elements C*D	-0.4	0.5444	-0.735	0.473 NS
**R^2 = 91.9% R^2 (adj) = 84.9%**<br>*** (significant <0.05) , NS (non-significant >0.05)**				

Supplementary Table S8. ANOVA for the quadratic polynomial model for atrazine degradation by *Klebsiella* sp. HA19

Source	Degree of freedom (DF)	Sequential sum of squares (Seq SS)	Adjusted sum of squares (Adj SS)	Adjusted mean squares (Adj MS)	F	P
Regression	14	1042.57	1042.57	74.469	10.75	0*
Linear	4	649.61	649.61	162.401	23.44	0*
Quadratic	4	114.41	114.41	28.603	4.13	0.017NS
Interaction	6	278.55	278.55	46.425	6.7	0.001*
Residual Error	16	110.84	110.84	6.928		
Lack-of-Fit	10	67.37	67.37	6.737	0.93	0.563NS
Pure Error	6	43.47	43.47	7.246		
Total	30	1153.41				
* (significant) NS(non-significant)						

Supplementary Table S9. ANOVA for the quadratic polynomial model for atrazine degradation by *Ochrobactrum* sp. A7

**Source**	**Degree of freedom (DF)**	**Sequential sum of squares (Seq SS)**	**Adjusted sum of squares (Adj SS)**	**Adjusted mean squares (Adj MS)**	**F**	**P**
Regression	14	864.98	864.98	61.784	13.03	0*
Linear	4	744.29	744.29	186.073	39.24	0*
Square	4	44.4	44.4	11.099	2.34	0.099NS
Interaction	6	76.29	76.29	12.715	2.68	0.054NS
Residual Error	16	75.87	75.87	4.742		
Lack-of-Fit	10	60.77	60.77	6.077	2.42	0.146NS
Pure Error	6	15.1	15.1	2.516		
Total	30	940.85				
* (significant)			NS (non-significant)			
